# Comparative bioacoustics of multiple eastern versus western songbird pairs in North America reveals a gradient of song divergence

**DOI:** 10.1371/journal.pone.0312706

**Published:** 2024-12-26

**Authors:** Lan-Nhi Phung, David P. L. Toews

**Affiliations:** 1 Department of Biology, Pennsylvania State University, University Park, Pennsylvania, United States of America; 2 Department of Biology, University of Rochester, Rochester, New York, United States of America; Rutgers The State University of New Jersey, UNITED STATES OF AMERICA

## Abstract

Vocalizations are one of the key premating reproductive barriers that could affect species formation. In song-learning birds, vocal traits are sometimes overlooked in species delimitation, as compared to morphological or plumage-based differences. In this study, we assessed geographic variation in songs of eight pairs of oscines on two scales: (1) comparing primary songs of species/subspecies pairs whose breeding grounds are eastern and western counterparts of each other in the continental North America, and (2) for each counterpart, identifying and comparing possible variation among their populations. We found that there were strong differences in the songs between eastern and western taxa, though the magnitude of that difference was not correlated to a mitochondrial DNA-based estimates of divergence. Additionally, we found that within-taxa geographic variation was not common in our focal taxa, beyond a single species (Townsend’s warbler, *Setophaga townsendi*). The result of this study provides a standardized, quantitative comparison of eastern and western songbirds, and serves as the foundation to explore the possible effectiveness of vocalizations as a reproductive barrier at this geographic scale.

## Introduction

The speciation of contemporarily diverse taxa like butterflies [1, 2], wood-warblers [3], poplars [4], and other taxa [5] has been associated with changes that occurred during the Pleistocene. This geological epoch was characterized by alternation between periods where ice sheets advanced and covered large land masses (i.e., glacials) and periods where these ice sheets receded (i.e., interglacials). Glacial refugia—contracted habitable regions where organisms retreated to for survival—isolated populations of species and potentially affected their divergence (and possibly subsequent introgression) upon secondary contact during interglacials [6]. The Laurentide ice sheet of North America has been shown to be one of the divides that separated refugia east and west of the Rocky Mountains, where contemporary species now also show repeated phylogenetic divergence in contact zones [6–8]. As a result of these processes, species that recolonized the continent post-refugia may have complex and diverse speciation outcomes. Studying the traits of contemporary species in the context of this glacial legacy is an important pursuit to understand the natural history of species and the divergence of lineages.

In avian species, one behavioral aspect that has received less careful attention in the context of this glacial history is vocalization. Historically, species and subspecies designations in birds have focused on morphological and plumage variants, yet over the past several decades researchers have increasingly recognized the value of considering vocal signals [9]. Studies have linked variation in vocalization and plumage with individual fitness and sexual selection [10]; these vocal traits have therefore been recognized as likely important reproductive isolation barriers in birds, and can therefore possibly provide insights about the extent to which gene flow affects the structure of populations. Moreover, in addition to the vertical inheritance across generations, vocal signals in song-learning birds can change via cultural mutation across a landscape—learning errors during the learning process may result in differentiating localized songs that are eventually passed onto the next generations [11, 12]; thus, it is relevant to assess these traits through a biogeographic lens to understand how songs vary geographically [13, 14]. Different geographic regions may promote divergence simply via isolation or restricted gene flow, or via the effects of adaptation to novel environments or different sexual selection pressures [reviewed in 10]. The result of cultural evolution of birdsong can be represented as geographic variants, which can be identified when the objective acoustic characteristics of songs vary more-or-less discretely across different geographic areas [15, 16].

In continental North America, studies have documented a specific pattern of song variation between eastern and western counterparts of closely related taxa, primarily divided by the Rocky Mountains. For example, Kroodsma [17, 18] described differences between vocalizations of the winter wrens and marsh wrens; both of which sing complex songs and show consistent and statistically detectable differences in song components, song types, and overall repertoires. The winter wrens of North America were eventually split into winter wren (*Troglodytes hiemalis)* and Pacific wren (*T*. *pacificus*) due to both bioacoustic and genetic evidence [19], while the marsh wren status requires additional genetic investigations.

Kroodsma [17] also noted that habitats in western North America are more heterogeneous. This means that there are smaller fragments of each species’ preferred habitat, which might prevent dispersal and result in stronger reproductive barriers in sympatry. Meanwhile in the east, larger fragments of suitable habitat may have allowed for a more gradual divergence and dispersal, as well as physical geographic isolation [17, 18]. This difference in habitat distribution, fragmentation, and history of isolation may affect the transmission of acoustic signals and the formation of dialects in the east and the west differently [9, 17, 20]. Specifically from the example above, western marsh wrens have quantifiably higher song complexity and repertoire size than eastern marsh wrens, despite presumably having the same learning capability [21]. Both species complexes do not hybridize in their sympatric ranges and, in this case, vocalization is likely an important reproductive barrier. Together, these studies show that the east-west distribution of the taxa coinciding with their vocalization differences might be a result of common divergence during geographic isolation and cultural evolution.

There have also been examples of geographic differences between populations within a single species in both eastern and western North America. One of the most prominent and thorough investigations is in white-crowned sparrows, *Zonotrichia leucophrys* [22–24]. Past studies of this system have focused on comparing differentiation of songs between populations with genetic and geographic structure, although the results have been mixed. Most recently, Lipshutz et al. [25] coupled genomic analysis with territorial playback experiment and found that two subspecies populations whose territories do not overlap also exhibit genetic divergence, as well as ecologically meaningful localized, fine-scale geographic variation in vocalization (i.e., dialects). Though not a direct correlation to genetic divergence, this study affirmed the role of dialects as a possible premating reproductive barrier as they might be driving the separation of populations and decreasing gene flow. In the east, mourning warblers (*Geothlypis philadelphia*) have been the focus of detailed research and appear to have four regional song types (i.e., regiolect; [26]) across the species’ breeding range based on syllable types and objective frequency and duration of songs, each type encompassing multiple populations [15].

Although the consistent vocal differences between eastern and western groups have been qualitatively described—like in the studies above—more systematic, comparative investigations are important to help reveal whether these differences vary primarily on an individual level, population level, and/or a large-scale, regional level, and whether this is an evolutionary phenomenon related to their historical geographic distributions. To do this also requires combining standardized measures of geographic differences in songs with time estimates of divergence, presumably derived from genetic data [6–8, 27–29]. One prominent example of divergence time estimation in avian species was by Weir & Schluter [30], who hypothesized that the speciation of boreal superspecies complexes were initiated and accelerated during the advancing of ice sheets, which forced their common ancestors into separate eastern versus western ecological ice-free refuges. They employed mtDNA cytochrome *b* sequences to calculate and compare coalescent timing for several pairs, which they found all fell within the Pleistocene and coincided with the timing of increased glaciation. This result implied that speciation of the examined species was likely initiated with glaciation as a geographic barrier, and potential trait divergence might have happened during this isolation. This divergence time estimation provided the basis for examining the vocal variation of species across the continental divide: if species divergence was a consequence of Pleistocene refugia, subsequent vocal divergence could be one of the key premating reproductive barriers that potentially promotes speciation and prevents hybridization upon post-refugia secondary contact.

The current study aims to describe the macrogeographic variation of songs in eight species pairs whose breeding grounds are eastern and western counterparts of each other, as a direct extension to Weir & Schluter [30] (Table 1). We assess the magnitude of song differences across two scales to explore variation both within and between taxa: (1) measure and compare the acoustic variation between eastern and western North American songbirds, and (2) identify acoustic variation within the range of each taxon and then compare any populations with detectable variation. These different scales characterize the pattern of variation and help determine whether regional differences exist, which will allow us to compare and detect potentially important vocal characteristics among taxa. Specifically, we predict that (a) there are objective, statistical differences in acoustic characteristics that distinguish populations across geographical areas on both scales, and (b) the magnitude of geographic differences between taxa would be larger than that of geographic variation within taxa. To estimate song divergence timing and determine whether that divergence correlates with the timing of Pleistocene events, we analyzed mitochondrial DNA (mtDNA) using phylogenetic analyses and coalescence time analyses. We chose mtDNA because it is maternally inherited and non-recombining and thus retains signals of historical isolation [31]. By contrast, differences in the nuclear genome can be eroded by contemporary gene flow [32]. This is particularly important for the taxa in question that, in many cases, have contact zones or hybrid zones where gene flow is a possibility. We predicted that if mtDNA divergence correlates with song divergence, it implies that song divergence might be consistent with Pleistocene-level isolation timing [30]; if there is no correlation, detectable song divergence might have evolved independently from mtDNA.

**Table 1:** Sampling details and acoustic measurements.

a.			
**Description of measurement**			**Measurement code**
Duration (s)			(1)
Peak frequency (Hz)			(2)
Minimum frequency (Hz)			(3)
Maximum frequency (Hz)			(4)
Number of repeats			(5)
Number of notes			(6)
Number of syllables			(7)
Up-slurred or Down-slurred (categorical, U or D)			(8)
Proportion of the song that is syllable A (%)			(9)
Measurements related to syllable A, B, C, …			(a), (b), (c), …
Measurements related to note 1, 2, …, of syllable A			(a1), (a2), …
Measurements related to note 1, 2, …, of syllable B			(b1), (b2), …
b.			
**Eastern counterpart**	**Western counterpart**	**Acoustic measurements**	
Eastern Meadowlark (*Sturnella magna*) *n* = 43	Western Meadowlark (*Sturnella neglecta*) *n =* 54	1, 2, 3, 4, 7, 1a, 2a, 3a, 4a, 6a, 1b, 2b, 3b, 4b, 6b, 1c, 2c, 3c, 4c, 6c (total: 20)	
Mourning Warbler (*Geothlypis philadelphia*) *n =* 50	MacGillivray’s Warbler (*Geothlypis tolmiei*) *n =* 50	1, 1a, 2a, 3a, 4a, 5a, 6a, 8a, 1a1, 2a1, 3a1, 4a1, 8a1, 1a2, 2a2, 3a2, 4a2, 8a2, 1a3, 2a3, 3a3, 4a3, 8a3, 1b, 2b, 3b, 4b, 5b, 6b, 8b, 1b1, 2b1, 3b1, 4b1, 8b1, 1b2, 2b2, 3b2, 4b2, 8b2, 9 (total: 41)	
Black-throated Green Warbler (*Setophaga virens*)—Type A *n =* 25	Townsend’s Warbler (*Setophaga townsendi*)—Type A *n =* 38	1, 1a, 2a, 3a, 4a, 5a, 1b, 2b, 3b, 4b, 5b, 1c, 2c, 3c, 4c, 5c, 1d, 2d, 3d, 4d, 5d (total: 21)	
Black-throated Green Warbler (*Setophaga virens*)—Type B *n =* 52	Townsend’s Warbler (*Setophaga townsendi*)—Type B *n =* 32	1, 1a, 2a, 3a, 4a, 5a, 1b, 2b, 3b, 4b, 5b, 1c, 2c, 3c, 4c, 5c (total: 16)	
Nashville Warbler–East (*Leiothlypis ruficapilla ruficapilla*) *n =* 50	Nashville Warbler–West (*Leiothlypis ruficapilla ridgwayi*) *n =* 50	1, 5a, 6a, 5b, 6b, 9, 1a1, 2a1, 3a1, 4a1, 8a1, 1a2, 2a2, 3a2, 4a2, 8a2, 1a, 2a, 3a, 4a, 1b1, 2b1, 3b1, 4b1, 8b1, 1b, 2b, 3b, 4b (total: 29)	
Yellow-rumped Warbler–East (Myrtle) (*Setophaga coronata coronata*) *n =* 73	Yellow-rumped Warbler–West (Audubon’s) (*Setophaga coronata auduboni*) *n =* 74	1, 1a, 2a, 3a, 4a, 5a, 1b, 2b, 3b, 4b, 5b, 1c, 2c, 3c, 4c, 5c, 7 (total: 17)	
Baltimore Oriole (*Icterus galbula*) *n =* 69	Bullock’s Oriole (*Icterus bullockii*) *n =* 65	1, 1a, 2a, 3a, 4a, 5a, 1b, 2b, 3b, 4b, 5b, 1c, 2c, 3c, 4c, 5c, 1d, 2d, 3d, 4d, 5d, 1e, 2e, 3e, 4e, 5e, 1f, 2f, 3f, 4f, 5f, 1g, 2g, 3g, 4g, 5g, 1h, 2h, 3h, 4h, 5h, 6 (total: 42)	
Wilson’s Warbler–East (*Cardellina pusilla pusilla*) *n =* 75	Wilson’s Warbler–West (*C*. *p*. *pileolata* & *C*. *p*. *chryseola*) *n =* 74	1, 1a, 2a, 3a, 4a, 5a, 1b, 2b, 3b, 4b, 5b, 1c, 2c, 3c, 4c, 5c, 7 (total: 17)	
Blue-headed Vireo (*Vireo solitarius*) *n =* 73	Cassin’s Vireo (*Vireo cassinii*) *n =* 60	1a, 2a, 3a, 4a, 1b, 2b, 3b, 4b, 1c, 2c, 3c, 4c, 1d, 2d, 3d, 4d, 1e, 2e, 3e, 4e (total: 20)	

(a) Acoustic measurements used in the study, accompanied by codes referred to in Table 1b. For example, the duration of note 1 of syllable B is codified as 1b1. If the syllable only contains one note, it will still be treated as a syllable. If the number stands alone (e.g., 1 instead of 1a), it is the measurement of the whole song. (b) Species pairs chosen for the study, with the sample size of each taxon and specific acoustic measurements employed for each pair. Sister species are marked in blue, closely related species are marked in green, subspecies are unmarked.

## Materials and methods

### Study species

**Fig 1:**
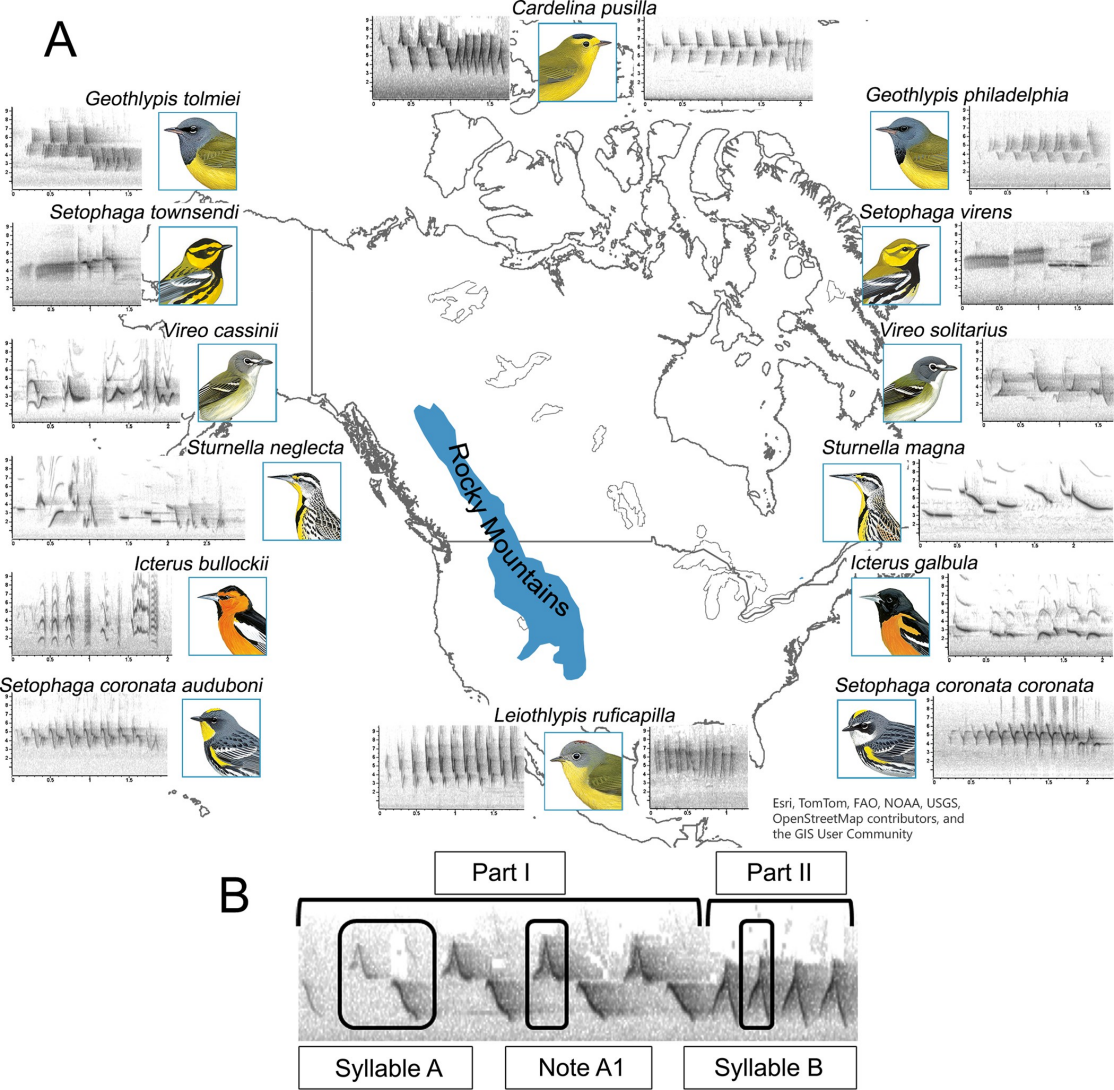
Study species description. (A) Continental North America divided by the Rocky Mountains. All study species are depicted alongside their songs (illustrations courtesy of Cornell Lab or Ornithology’s Birds of the World). (B) An example of an eastern *Leiothlypis ruficapilla* song, showing the definition of acoustic components measured in the current study: syllable vs. note. This map was made with Natural Earth (free vector and raster map data at naturalearthdata.com) and ArcGIS Online basemap [41].

We selected eight pairs of closely related migratory passerines based on previous molecular phylogeny and coalescent time analysis, whose breeding grounds are in continental North America [30, 33–37]. Each pair consists of an eastern taxon and a corresponding western taxon (i.e., “counterparts”), all listed in Table 1. The chosen boundary between eastern and western taxa is the Rocky Mountains because phylogeographic breaks along this mountain range are found in many species, specifically avian species where they shared recolonization routes post-glaciation [6]. These closely related pairs were chosen so that each taxon is the closest eastern and western representative of their phylogenetic group; three are sister species, two are closely related species, three are subspecies (Fig 1A, Table 1), and many hybridize. Among the eight pairs, *Setophaga virens* and *Setophaga townsendi* each have two known song types, which we identified and compared separately [38, 39]. *Cardellina pusilla* has three subspecies, two of which *C*. *p*. *pileolata* and *C*. *p*. *chryseola* we treated as a single group (western) in comparison to *C*. *p*. *pusilla* (eastern) due to genetic divergence [11, 40].

### Measuring songs

We obtained all song recordings from the Macaulay Library (http://macaulaylibrary.org). We filtered and chose only high-quality recordings that were recorded during the breeding season of most recent years (May and June of 2015 to 2021) distributed throughout the known breeding range of each counterpart to capture song variations that might track along those landscapes. We also chose only primary songs—the song type chiefly used by male birds to communicate territory and mate attraction—based on each taxon’s known natural history and the metadata from the Macaulay dataset. For taxa with widespread female singing behaviors like *Icterus* taxa and *Sturnella* taxa, analyses with male-only songs yielded the same pattern of clustering (Fig E in S1 File); therefore, we included songs identified in the metadata as “female” or “unknown” to increase the sample size and to include female songs. High-quality recording was determined first by filtering for only recordings with 3 stars and higher rating via eBird’s user-rated 5-star scale, and then by objective assessment during the scoring process. That is, if recordings contain too much background noise or lack of power when viewed as spectrograms (i.e., too faint that the measurement cannot be extracted), they were eliminated and replaced with other recordings. We also excluded recordings from known hybrid zones and used only recordings with confirmed identities by the Macaulay metadata to avoid potential hybrid songs (e.g., excluded *Setophaga coronata* from eastern British Columbia and western Alberta region) and recordings too close to the edge of each range to avoid the confounding impact of the interaction between songs when different species are in sympatry [42]. The sample size varied across pairs within the range of 51–83 recordings per counterpart (Table 1). We collected measurements manually using Raven Pro 1.6 [43] from each individual’s most complete song. These measurements included: duration of song (s), number of unique notes/syllables, whether the song is up-slurred or down-slurred (categorical, U or D), and up to 5 variables measuring each note/syllable such as duration (s), peak frequency (Hz), minimum frequency (Hz), maximum frequency (Hz), and number of repeats for each note/syllable. This method of assessing acoustic characteristics was adapted from Kenyon et al. [42], which was chosen for its versatility and extensiveness appropriate for this set of taxa. The set of measurements for each pair is differently chosen based on the characteristics of the songs (Table 1); for example, the song of *C*. *pusilla* has two syllables, each repeated multiple times, so the set of measurements would include measurements of each syllable and the number of repeats, while *I*. *galbula* has many syllable types being sung not in one order, so the set of measurements would include only duration of each syllable and the number of repeats without an overall duration. Here we defined a syllable as one uninterrupted unit whose components always accompanied each other when repeated; a note is defined as an individual component separate from other components, multiple of which could form a syllable or a whole song (Fig 1B). Both are determined visually via spectrograms. For songs that have more consistent structure (e.g. *Setophaga*, *Cardellina*, Fig 1A), it was more appropriate to assess syllables as an entity in addition to notes, while for songs that are structurally more varied (e.g. *Vireo*, *Icterus*, Fig 1A) an assessment of individual notes was more meaningful in capturing the bioacoustic range of the taxa. We extracted maximum and minimum frequency using Raven’s Peak Frequency Contour (PFC) tool set, specifically PFC Max Freq and PFC Min Freq. These features allowed us to gain an objective detection of sounds comparing to the manual selection offered by basic features, which is typically subjected to inter-rater variability [44].

### Statistical analysis of songs

We implemented three independent statistical analyses to assess the geographic variation in acoustic characteristics of songs. The two clustering analyses are done for each counterpart to assess within-taxa variation, and then for comparison between the counterparts of each pair to assess between-taxa variation, while Δ*p* was used to assess between-taxa variation only. All measurements collected from Raven Pro 1.6 as song features were used for quantifying song divergence via both all three methods. First, we used partitioning around medoids (PAM), a robust k-means clustering method, by running two functions in the R package ‘cluster’: ‘daisy’ to calculate dissimilarity matrices using Gower’s distance, and ‘pam’ to cluster data into ‘k’ using the matrices generated by ‘daisy’ [45]. As opposed to the Euclidean distances that are the root sum-of-squares of differences typically used for numerical-only datasets, Gower’s distance first standardized each variable so that the dissimilarity scores are within the range of [0,1], which makes it possible to measure dissimilarities between categorical values where it is either 0 (no difference) or 1 (different) [46]. This feature allowed for mixed datasets of numerical and categorical data as well as “missing data”, because the variation in number of notes and syllables occasionally caused our datasets to be uneven; the ‘daisy’ algorithm omits the missing data from the dissimilarity calculations so that the scores only contain the individual’s available song features. To determine k for each PAM analysis, we used silhouette width—a metric used in clustering analyses to assess how similar within-cluster data points are to each other comparing to the neighboring cluster. We calculated silhouette width for k = 2–8 using the built-in ‘sil_width’ function from the package and chose the highest value, which signifies the best fitting k. Because PAM generated dissimilarity matrices for each comparison, we also assessed the magnitude of song divergence: (1) within the eastern taxon, (2) within the western taxon, and (3) between the eastern vs. western taxa. We used permutation test for each pair to compare whether the dissimilarity of between-taxa is greater than within-taxa, accounting for the resampling of each individual during the generation of the dissimilarity matrix. We used the R package ‘coin’ to generate permutation tests [47], particularly an approximative reference distribution (Monte Carlo) with 10,000 replicates. We built a model with dissimilarity score as the variable of interest assigned to one of the three categories of comparison: (1) within the eastern taxon, (2) within the western taxon, and (3) between the eastern vs. western taxa. Individuals were then resampled 10,000 times to generate a distribution of the summary statistic under the null hypothesis that there is no difference between populations. Finally, the observed summary statistic from the actual populations was compared to the distribution of summary statistics from the permutations—*P*-values calculated from these comparisons was adjusted with post-hoc false discovery rate. These analyses allowed us to assess whether further geographic distance (between-taxa) correlates with more diverged songs.

Second, we used the ‘PCAmixdata’ package to perform principal component analysis (PCA), another common clustering method [48]. Similar to PAM, ‘PCAmix’ calculated the principal components and loadings for our mixed and uneven datasets to determine the pattern of song divergence and if so, which variables distinguish them. PCAmix also replaced missing data with means for quantitative variables and with zeros for categorical variables automatically before calculations. Graphical presentation of PCA results and interpretation used chiefly PC1 and PC2 because they explain the majority of variation in each taxon (see Result; Table 2; Tables C & D, & Fig A in S1 File).

**Table 2:** The statistical result of PCA, PAM, and all-trait Δ*p* for each between-taxa comparison.

	PCA		PAM			Δ*p*
	PC1 (%)	PC2 (%)	Dim1 (%)	Dim2 (%)	k	
*S*. *magna* vs. *neglecta*	42.27	11.79	61.8	20	2	187.19*
*G*. *philadelphia* vs. *tolmiei*	21.93	13.33	42.1	24.2	2	166.31*
*S*. *virens* vs. *townsendi*	A: 31.75	A: 14.99	A: 61.6	A: 49.1	A: 2	A: 136.42*
	B: 21.97	B: 21	B: 16.5	B: 14.6	B: 2	B: 117.70*
*L*.* r*. *ruficapilla* vs. *ridgwayi*	19.09	11.01	25.7	20	2	128.18*
*I*. *galbula* vs. *bullockii*	10.74	9.602	36	18.9	3	118.51*
	Log: 18.4	Log: 14.5				
*C*. *p*. *pusilla* vs. *pileolata* & *chryseola*	14.43	13.03	42.7	26.6	2	115.60*
*S*. *c*. *coronata* vs. *auduboni*	19.64	13.18	44.7	24	2	81.07*
*V*. *solitarius* vs. *cassinii*	12.21	11.65	36	12.1	1	71.93*

Magnitude of divergence based on PCA and PAM are placed in the descending order of Δp values. *: *P* < .05

For consistency and comparative purposes, the tools were used on all counterparts regardless of whether their dataset contains categorical variables. Both tools allowed us to assess acoustic differences without *a priori* eastern versus western grouping and assess whether these differences match the known geographic ranges, as well as detecting any potential within-taxa variation. For both PAM and PCA, we expected to observe clear clustering of groups whose acoustic characteristics are distinguishable from each other, as well as some high-loading variables that drive the differences. Using the clusters blindly determined by PAM, we mapped the locality of each singer onto their respective range maps produced by BirdLife International [49] to visually identify geographic structure. Geographic variants would be identified if we observed (1) clear clustering of groups as shown in graphical representation of PAM and PCA and (2) these groups can be distinguished by their geographic locality.

Finally, we quantified the magnitude of divergence of each pair using Δ*p*–a metric of phenotypic distance that allowed for multi-trait comparison between two populations [50]. Due to the nature of song diversity among the study species, it was not possible to compare each measurement directly (e.g., the difference in note A between *Vireo solitarius* and *V*. *cassinii* would be different from the difference in note A between *S*. *c*. *coronata* and *S*. *c*. *auduboni*); therefore, we calculated Δ*p* as a standardized “divergence score” to compare the pairs to each other. Δ*p* is a non-parametric distance measure calculated based on a joint cumulative distribution function (CDF), where a joint CDF was first calculated for each raw song feature across both populations. Then, for each population, a median percentile coordinate in the dimension of each feature was determined relatively to this CDF. Repeating this calculation for the remaining song features, the consequent Δ*p* is the Euclidean distance between two populations calculated using the median percentile coordinates of all song features. Δ*p* are relative values to the data set, and it is robust against unequal variance and sample sizes and considerable missing data, particularly compared to Hedge’s *g* [50]. We executed the MATLAB script developed and provided in the original paper (http://sourceforge.net/projects/deltap/files/) to import the data and calculate Δ*p*, Hedge’s *g*, and descriptive statistics for each song feature and the pairwise whole-population Euclidean distance. The reported Δ*p* in Table 2 that we used as “divergence score” is the whole-population distance.

### mtDNA phylogenetic analysis

Publicly available cytochrome *b* sequences of all species were acquired from NCBI’s GenBank and used to generate a rooted timetree using the maximum-likelihood approach (Table 1). Using MEGA11, an initial phylogenetic tree was generated using neighbor-joining method, then variants of this tree were created using nearest neighbor interchange (NNI), and finally a best fitting tree was computed based on the general time reversible (GTR)-gamma model [51]. With this maximum-likelihood phylogenetic tree, the function “Compute TimeTree” in MEGA11 was used to estimate divergence time using the “RelTime-ML” analysis, and the analysis preference were applied as suggested by Mello (2018) [52]. As calibration information is not required by the RelTime method, MEGA11 calculated a relative time scale, and this scale was used in the resulting timetree. We used a sequence of the peregrine falcon (*Falco peregrinus*) as the outgroup. These estimates of mtDNA divergence between each species pair were used as a proxy for the timing of Pleistocene splitting events [30]. Spearman’s rank correlation statistic was used to assess the relationship between mtDNA divergence and song divergence score Δ*p*.

## Results

### Song divergence within taxa

Overall, we did not find any obvious within-taxa geographic variation (Table A in S1 File). We found statistical differences in acoustic characteristics that distinguish populations based on k>1 clusters in all counterparts (i.e., not all songs could be lumped into a singular group) except for *S*. *c*. *auduboni*, but these clusters mostly did not relate to variation in locality. For example, *C*. *p*. *pusilla* has a dominant song sung by most individuals and a variant song that is sung less frequently (12 out of 75 individuals), both distributed throughout its eastern range. Similarly, *S*. *c*. *coronata* has a variant song different from a dominant song, sung by 22 out of 73 individuals but was not geographically related. *I*. *bullockii* shows two equally prevalent song variants without a geographically related distribution across its range.

Some Western counterparts exhibited clustering that might be geographically structured. *C*. *p*. *pileolata* and *chryseola* were treated as a single group (western) in this analysis, but PAM estimated that the inland song type matches *C*. *p*. *pileolata* range and the coastal song type matches *C*. *p*. *chryseola* range. However, these differences were not large (PC1 = 15.55%). Similarly, PAM also detected a coastal song and an inland song in *G*. *tolmiei* (PC1 = 18.47%).

The clearest localized geographic variant was detected in song type B of *S*. *townsendi*: a cluster of individuals in lower mainland British Columbia (Vancouver area) was distinguished from the rest of the range (PC1 = 24.14%); in other words, the song type throughout the rest of the range is the dominant song while these 8 Vancouver individuals sang a variant. An additional 13 songs in this British Columbia region (3 type A, 10 type B) were added to the data set to increase the confidence of these findings, to which we found the same group determination in both PAM and PCA (PC1 = 26.69%), as well as a significant t-test for the highest loading variable of that grouping: maximum frequency of syllable C; *t*_23_ = -5.14, *P* = 3.11x10^-5^ (Fig D in S1 File).

### Song divergence between taxa (Eastern vs. western)

**Fig 2:**
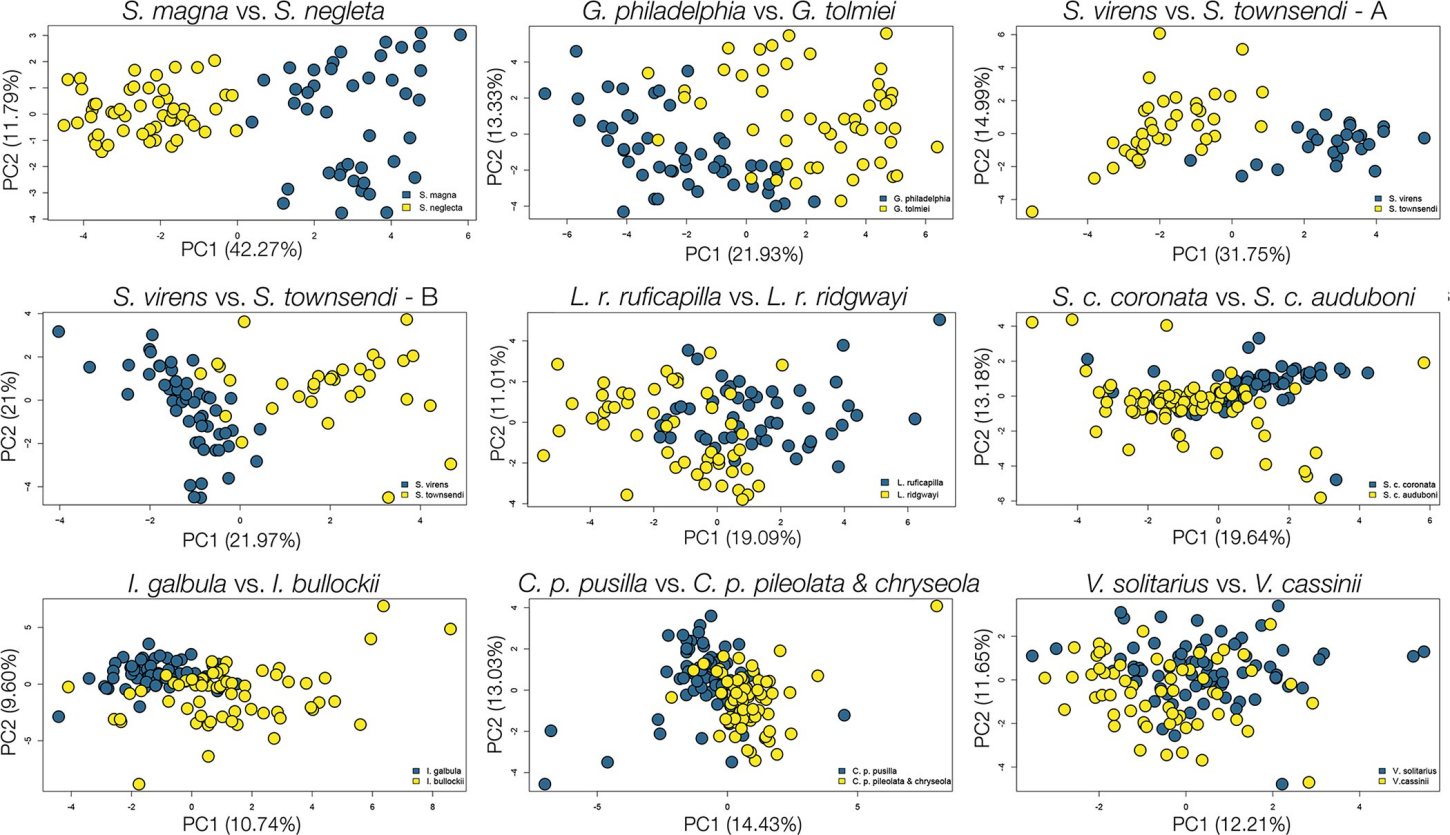
Principal component analysis (PCA) for each species pair. This figure includes analyses for both song types of *S*. *virens* and *S*. *townsendi*. Individuals of Eastern counterpart are shown in blue, individuals of Western counterpart are shown in yellow.

**Fig 3:**
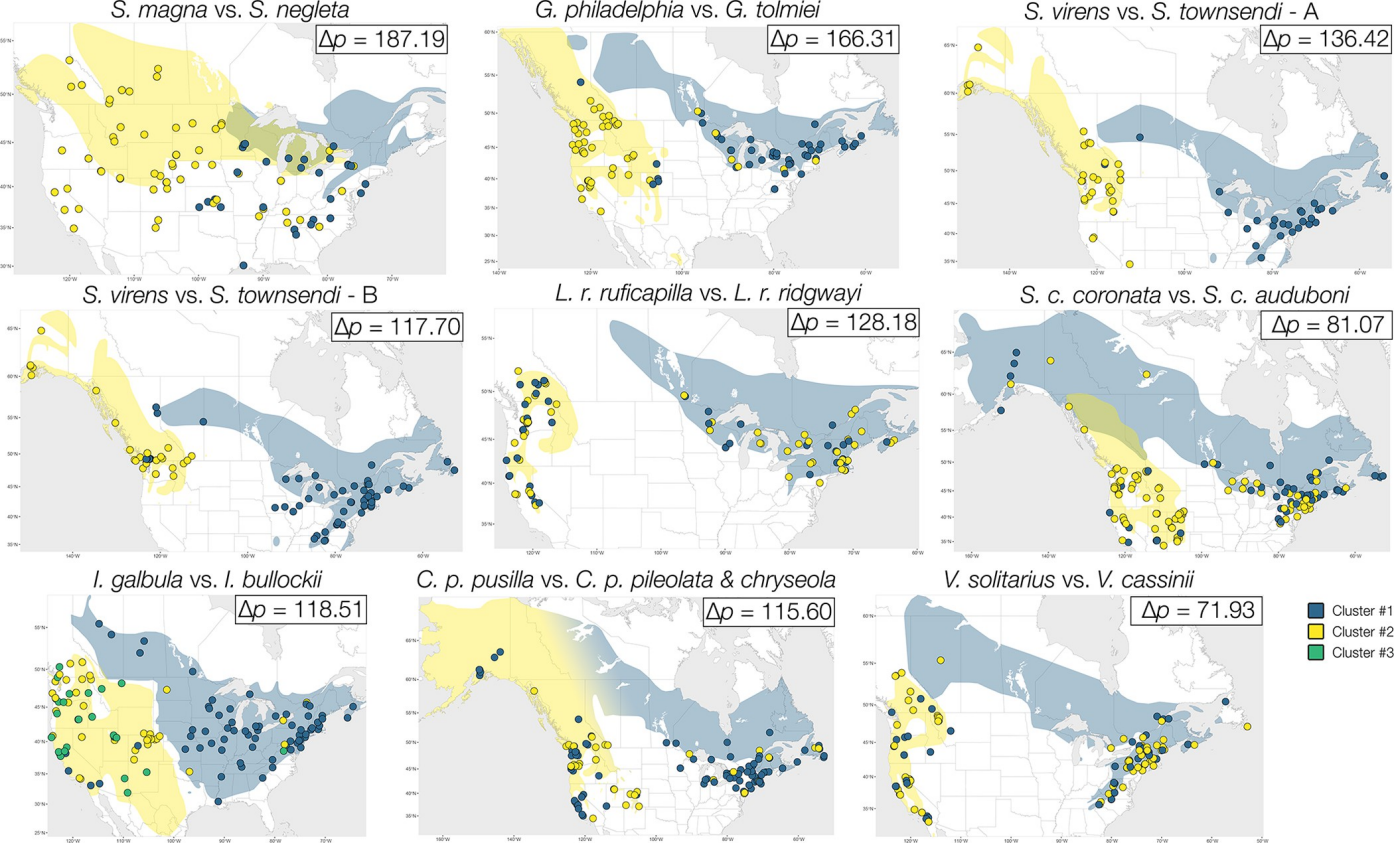
Map of geographical variation in songs of eight closely related avian pairs based on partitioning around medoid (PAM) analysis. This figure includes analyses for both song types of *S*. *virens* and *S*. *townsendi*. The polygons underneath the data points are range maps of each counterpart: eastern ranges are in blue, western ranges are in yellow. Taxa of the same pair share the same shape on each map; eastern individuals are in blue and western individuals are in yellow. This map was made with free basemap available via geoBoundaries [53], an open database of political administrative boundaries. CC BY 4.0.

**Fig 4:**
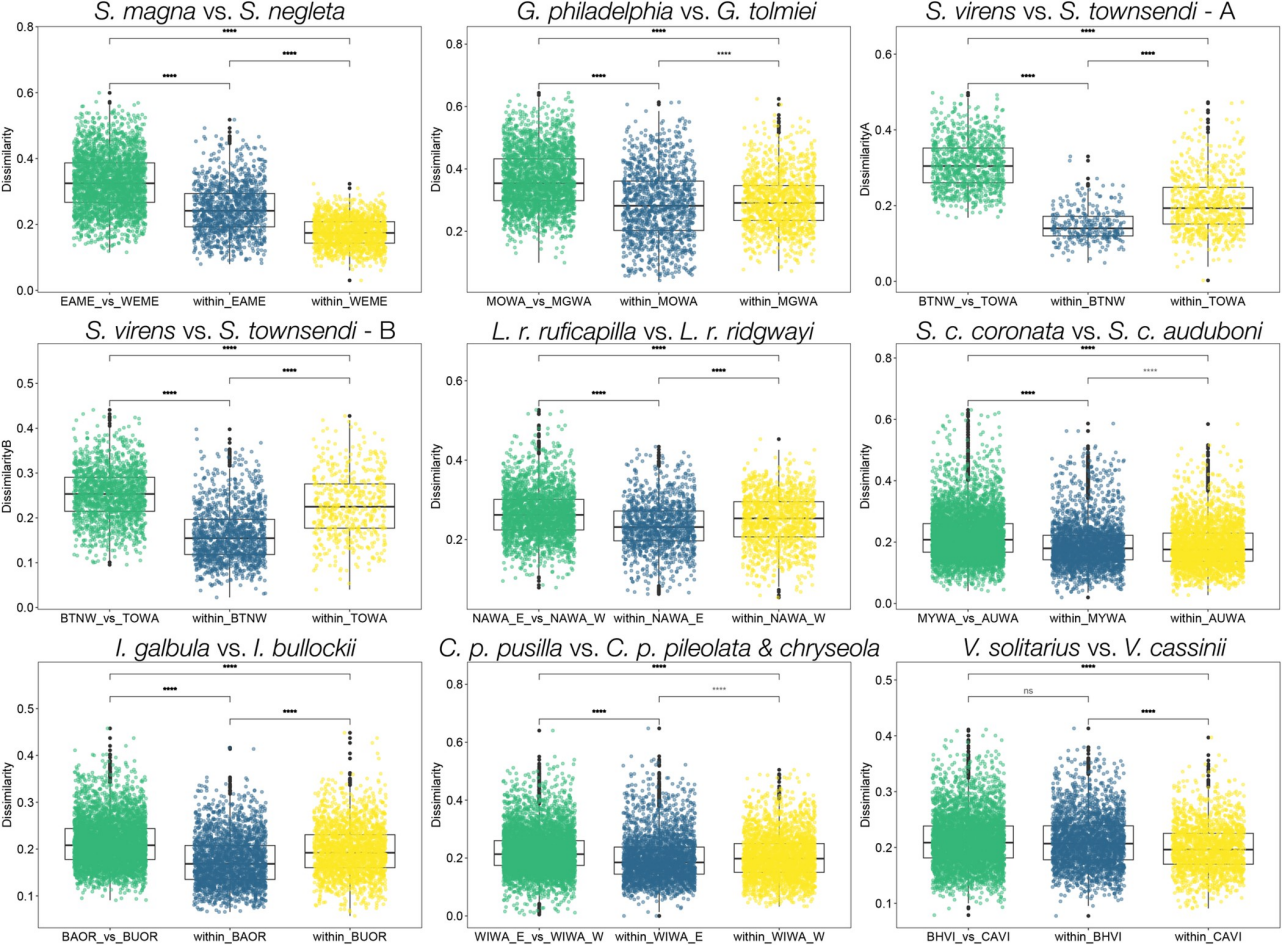
Magnitude of differences in within- and between-taxa comparisons. Comparison of the magnitude of differences (i.e. dissimilarity) among within eastern counterpart (blue), within western counterpart (yellow), and between eastern vs. western counterpart (green) based on dissimilarity scores for each species pair. Each point is an overall dissimilarity score based on all song features, which represents a comparison between two individuals. Thus, blue points are comparisons among individuals of eastern counterparts, yellow points are comparisons among individuals of western counterparts, and green points are comparisons among individuals of both groups. Significance values shown are from permutation: *: *P* ≤ 0.05, **: *P* ≤ 0.01, ***: *P* ≤ 0.001, ****: *P* ≤ 0.0001. Bolded values are those remain significant following Bonferroni correction, *P* ≤ 0.00185.

The divergence between counterparts of each pair is reported in Table 2. Overall, each pair’s songs fall into two PAM clusters and variation is primarily explained by the first two dimensions of PCA, except for the *Icterus* pair which have songs that best fit best into 3 clusters (Fig 2, Table 1, Figs A, C in S1 File). The dissimilarity statistics PAM generated for within- and between-taxa is shown in Fig 4; for the majority of comparisons, the dissimilarities between taxa are significantly larger than the dissimilarity within each taxon, tested with permutation analyses with Bonferroni correction for significance criteria, *P* ≤ 0.00185 (Fig 4, Table B in S1 File). The groupings determined by both PAM and PCA correlated with the biogeography of each pair, though to varying degrees. The east-west divide is very distinct in *S*. *magna* vs. *neglecta* (Δ*p* = 187.19), *G*. *philadelphia* vs. *tolmiei* (Δ*p* = 166.31), and *S*. *virens* vs. *townsendi* (Δ*p* = 136.42 for type A, 117.70 for type B), within all of which the groupings of most individuals match their locality (Fig 3). This divide is less prominent for the rest of the pairs, where *L*. *r*. *ruficapilla* vs. *ridgwayi* (Δ*p* = 128.18) and *S*. *c*. *coronata* vs. *auduboni* (Δ*p* = 83.66) showed moderate geographic divergence (Fig 3), and *I*. *galbula* vs. *bullockii* (Δ*p* = 118.51), *C*. *p*. *pusilla* vs. *pileolata* and *chryseola* (Δ*p* = 115.60) and *V*. *solitarius* vs. *cassinii* (Δ*p* = 71.93) showed low geographic divergence (Fig 3). Interestingly, the stratification of Δ*p* did not always match the categorization by PCA and PAM. Although the highly diverged pairs have the highest Δ*p* values and the least diverged pair has the lowest Δ*p* value, the magnitude of divergence of the “moderate” pairs are incongruent. For example, *I*. *galbula* vs. *bullockii* pair have low PC1 and 2 values and moderate PAM percentage while having high Δ*p* values; the *C*. *p*. *pusilla* vs. *pileolata* & *chryseola* pair have very low PC1 and 2 and quite high PAM percentage and have high Δ*p*. We also found that the differences between the sets of measurements for each pair did not affect the divergence score measured as Δ*p* values; for example, the *Icterus* pair has 42 measurements, yet its Δ*p* value is similar to that of the *Leiothlypis* pair which only has 29 measurements.

### Correlation between mtDNA divergence and song divergence

**Fig 5:**
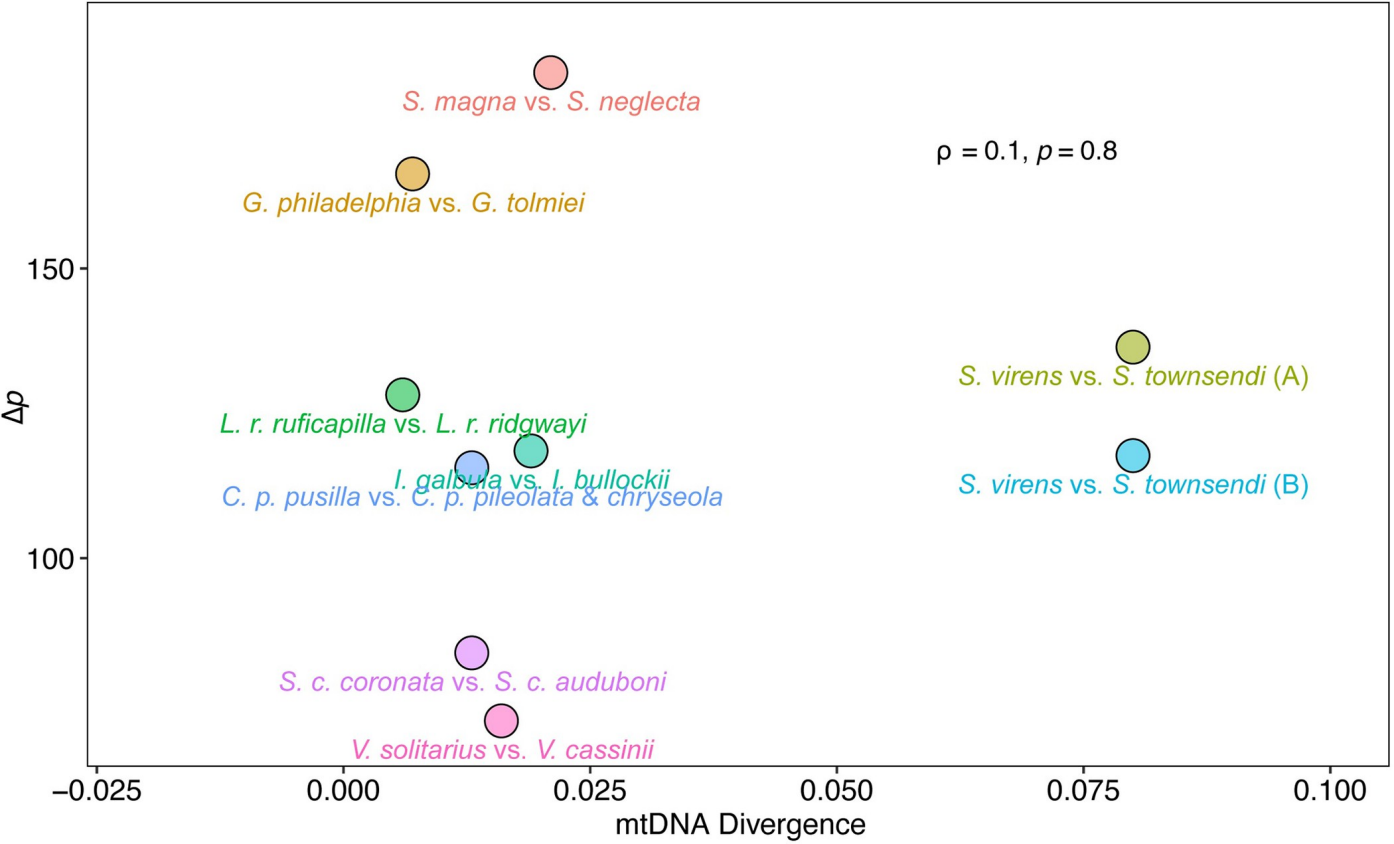
Correlation between coalescent estimated mtDNA divergence and song divergence score Δ*p* of all species.

The time-estimated mtDNA divergence between taxa of each pair ranges between 0.005 to 0.021 in relative time (Fig B in S1 File). When mapped against the Δ*p* values as shown in Fig 5, no obvious correlation was detected (Spearman’s ⍴ = 0.1, *p* = 0.8). This suggests that the degree of mtDNA divergence is not predictive of song divergence, even though a spectrum of song divergence was observed across all pairs.

## Discussion

Here we present a systematic analysis of song divergence in various oscines of North America using three independent metrics, which build upon and complement previous studies on each species pair. We found evidence of differences between east-west distributions and vocal differences on a continental scale—considered between-taxa geographic differences—where closely related species differ in their vocalizations. The magnitude of these differences ranges from high, moderate, to low, depending on the species pair. Although there were many shared variables in each pairwise comparison, we did not detect any acoustic features that consistently contribute to the overall acoustic differences across the pairs (i.e., no variable had consistently high PC loadings in multiple pairs; Table D in S1 File). We also tested the relationship between the magnitude of these song differences with divergence time at *cytb* and found no correlation between the two. This lack of relationship implies that the differences between pairs in their song is not entirely predictable based on their divergence time, at least based on mitochondrial dating. This means that song differences may change more rapidly or idiosyncratically than is captured by isolation time alone, but also suggests that markers from detailed, whole genome data might be more useful to better characterize historical differences that might relate to song variation. We also found that for each pair, the magnitude of song differences between eastern-western counterparts are consistently larger than the magnitude of song differences within each counterpart—this affirms that the comparisons we made captured the song difference relative to each pair, and that our method of determining geographic variation based on range maps and individual locality is sufficient in revealing the geographic pattern (or the lack thereof). On a smaller geographic scale, we did not find many potential within-taxa acoustic variants that separate adjacent populations of the same taxa, except for a song type of one *S*. *townsendi* population. Although no common pattern of variation was observed for all taxa, our findings further suggest that song as a trait varies across geographical space, which could be a precursor for the formation of well-defined regiolects and dialects.

The lack of clear within-taxa geographic differences was an unexpected pattern, particularly due to the previously reported complex geographic variation in some prominent examples (e.g., *G*. *philadelphia*; 15). We did not observe strong geographic bioacoustic differences within the taxa presented in the current study; however, it is possible that this is because there were slight differences in the set of measurements; for instance, Pitocchelli [15] stereotyped the primary syllable and its variation while we did not. Further playback studies will be needed to assess song discrimination to determine the importance of subtle differences between within-taxa populations. The sole potential local geographic variant detected in one of the two song types in *S*. *townsendi* could serve as a case study for this work—our results suggest a separation between the highly populated area around the city of Vancouver versus the natural areas throughout the rest of its range. Specifically, this difference was mostly driven by the maximum frequency of the third syllable (also the maximum frequency of the whole song), of which the putative Vancouver local geographic variant is significantly higher (Fig D in S1 File). Different habitats associated with vocalization divergence has been documented in previous studies of oscines, mostly related to vegetation density, which affects the transmission of vocalization [9, 20, 54]. Several species have been shown to have gradually changed their vocalizations to sing louder in the urban landscapes compared to forests [55]. Moreover, urban areas have been shown to have higher attenuation and reverberation compared to rural areas and urban songs tend to have short whistles, faster trills, and narrower bandwidth, which are evidence that the landscapes might affect both sound transmission and the song characteristics themselves [56]. Counter to our findings in *S*. *townsendi*, Phillips and colleagues [56] found that the city landscape correlated with lower maximum frequency. However, the difference in *S*. *townsendi* is likely between urban/suburban vs. mountain habitat, which might differ from the “rural” landscape outlined in [56]. Recent work by Ore and colleagues [57] identified a similar geographic pattern where Vancouver *S*. *townsendi* individuals sang a geographically localized type B song (Type I in that study), which confirms and extends our own findings. We suggest future work could benefit from additional sampling as well as playback experiments in *S*. *townsendi* to fully assess the possible sound transmission difference in these different landscapes and understand the development of this local geographic variant in *S*. *townsendi*.

In comparing geographic differences between eastern-western taxa, the divergence score *Δp* presented a spectrum of acoustic divergence: the three pairs with the highest *Δp* (*S*. *magna* vs. *neglecta*, *G*. *philadelphia* vs. *tolmiei*, and *S*. *virens* vs. *townsendi*) also have the most variance explained by the first two dimensions in both PCA and PAM (Table 2). Similarly, all three metrics agreed on the low divergence across their geographic range for the least diverged pair *V*. *solitarius* vs. *V*. *cassinii*. We consider the other four pairs (*L*. *ruficapilla* subspecies, *C*. *pusilla* subspecies, *S*. *coronata* subspecies, and *Icterus* species) to have moderate to low song divergence, as the methods gave discordant results on where they would fall on the divergence spectrum (Table 2, Figs 2 and 3). Our findings are consistent with the current knowledge that *S*. *magna* vs. *S*. *negleta*, *G*. *philadelphia* vs. *tolmiei*, and *S*. *virens* vs. *townsendi* are highly diverged genetically and show strong geographic structure across their breeding ranges [33, 35, 36, 58]. Although we did not examine reproductive isolation in this study beyond reasonable speculation, the systematic and comparative quantification of song divergence presented in this study contributes to the current knowledge of each taxon and may serve as the basis for subsequent studies such as playback studies to further examine the reproductive isolation via songs in these species.

Hybridization and the maintenance of hybrid zones have provided some interesting insights into the dynamics of song variation between eastern and western taxa. In the case of *S*. *virens* vs. *S*. *townsendi*, previous studies found that song types match species membership in allopatry, and individuals in the hybrid zones would sing one song or the other regardless of whether they are genetically “more *S*. *virens*” or “more *S*. *townsendi*” [42]. Reciprocal playback experiments in the same study showed that allopatric birds have a stronger territorial response to conspecific songs and not heterospecific songs, while in the hybrid zone there were no observed differences in responses. This implies that song may be a strong reproductive barrier in allopatry, but this breaks down in the hybrid zone, where it is most relevant [42]. In contrast, while having a similarly narrow zone of extensive hybridization, *S*. *philadelphia* and *S*. *tolmiei* hybrid song is a blend of the two allopatric songs that differed from both, and the song types in the hybrid zone are not predictive of species membership determined by genetic markers [59, 60]. The implication from this study was also that song was not a reliable reproductive barrier for *S*. *philadelphia* and *S*. *tolmiei* hybrids, due to the lack of song learning discrimination. In both cases, singing and learning songs that are not conspecific does not appear to contribute to selection against hybrids, but the influence of these song patterns within the hybrid zones also does not seem to go beyond the zones and affect allopatric birds. Since our analysis excluded potential hybrid songs, it is consistent with the literature that we found high song divergence between these taxa in allopatry, implying that these taxa have a strong barrier via songs despite maintaining hybrid zones.

The *S*. *coronata* subspecies pair and *Icterus* species pair are both previously shown to hybridize, despite evidence showing a strong reproductive barrier in plumage and a weak barrier in songs, and that genetic divergence is significant in both pairs [35, 61–63]. The species status of both systems are highly debated because both actively hybridize, but only in their narrow hybrid zone—the rest of the continental range remains differentiable in both plumage and genetics. The songs between counterparts in each pair also diverged per our findings here, though the magnitude of the differences is smaller compared to other hybridizing *Setophaga*, as discussed above. Previous playback experiments for *S*. *coronata* subspecies also suggest that song is not a reliable reproductive barrier both within and outside of the hybrid zone [61], whereas this is unknown for *Icterus* species due to the lack of playback experiments. Additionally, recent genomic and plumage analyses of *Icterus* species suggest a gradient of genomic admixture within the hybrid zone which correlates with the phenotypes of hybrid plumages. This implies moderate reproductive isolation within the hybrid zone, possibly due to an unknown form of selection against hybrids [64, 65]. For both pairs, there is substantial genomic differentiation between them, yet the precise phenotypes relating to those genetic differences are still unknown. Thus, while speculative, it is possible that one or some of these genomic regions might affect their vocalizations indirectly, such as via beak morphology or song-learning ability, and subsequently alter hybrid fitness [66, 67]. It is also notable that although the *Icterus* taxa are currently considered two species, *S*. *coronata coronata* and *S*. *c*. *auduboni* are currently not.

*C*. *pusilla* subspecies is the only pair in this study that has a completely contiguous breeding range; interestingly, there has been substantial evidence of genetic divergence between eastern and western subspecies despite not exhibiting stark differences in plumage and morphology [40, 68–70]. The eastern subspecies *C*. *p*. *pusilla*—distributed throughout the large fragments of suitable habitat of the continental east—forms a genetically cohesive group with minimal geographic variation within and is distinct from the western subspecies [70]. Although we used songs from both western subspecies to represent a sole “western” group, we still detected some geographic structure of song variation that might be concordant with the distributions of *C*. *p*. *pileolata* and *C*. *p*. *chryseola*, but a larger sample size is needed to confirm this pattern (Table A in S1 File). Consistent with genetic studies, the difference between these western subspecies is seemingly smaller than the difference between the eastern and the western groups according to dissimilarity score generated by PAM analysis (Fig 4, Table B in S1 File). Moreover, individuals of the western group whose distribution matches *C*. *p*. *pileolata* always grouped with the eastern populations in the eastern vs. western song comparison (Fig 3). We suspect due to the continuous distribution in the north, but not throughout the central US, the eastern song might have been transmitted and blended into songs along the coast, while the western inland song stays moderately different. Moreover, since the population boundaries between the *C*. *pusilla* subspecies are not known, we could not confirm the identity of the individuals we chose to analyze along the proposed Rocky Mountains boundary, which might have made our analysis less accurate. Further investigation into song recognition is needed between the subspecies to make conclusions about song variation.

For the non-hybridizing pairs, we draw a contrast between the most and the least diverged songs: the *Sturnella* and the *Vireo* pairs, respectively. Beam and colleagues [58] suggested that eastern *Sturnella magna* and western *S*. *neglecta* are highly diverged both acoustically and genetically from each other, as well as from the southwestern Lilian’s subspecies (now split and renamed as *S*. *lilianae*). Given the virtually identical plumages and the additional evidence of substantially diverged songs we provided here, yet a lack of hybridization, future playback experiments will be crucial to assess whether song would play a role in species recognition between *S*. *magna* and *S*. *neglecta*. In the least diverged pair, *V*. *solitarius* and *V*. *cassinii*, all three metrics PCA, PAM, and Δ*p* agreed on their low song divergence across their geographic range. Coupled with low plumage difference between them, this outcome raises questions about which reproductive barrier is most important for the species boundary, since the two common mechanisms seem to be weak. mtDNA evidence justified their split into separate species [34, 71, 72], but since then there has been much back and forth debate on the phylogenetic relationship within the vireo complex that also includes *V*. *plumbeus* of the western interior.

This study allows us to assess the unique situation of the *L*. *ruficapilla* subspecies, which have allopatric ranges with a presumably strong dispersal boundary separating them (i.e., the Great Plains). *L*. *r*. *ruficapilla* and *L*. *r*. *ridgwayi* have diagnosable differences in plumage, behavior, and calls [73]; here, we found that songs also differentiate them, though it is unclear whether these acoustic differences are the result of the range separation, or whether stochastic cultural mutations had occurred and spread before range separation. Allopatric species pairs have overall greater plumage and song divergence compared to sympatric pairs [74]; this is potentially because allopatric species tend to inhabit different habitats and sympatric species tend to inhabit similar habitats, which can be important in the context of plumage perception and sound transmission [9, 75]. Together with previous knowledge, the song divergence we found here is a starting point for further investigation regarding the species status of *L*. *ruficapilla* subspecies, as the magnitude of divergence between their songs are higher than pairs currently considered different species (e.g., *Icterus* and *Vireo* species). *L*. *ruficapilla* is also the least studied of all taxa in the current study: the most recent genetic work examined only the mtDNA, which showed two distinct haplotypes corresponding with the east-west grouping [76]. It is crucial for future studies to assess the genetic difference and song discrimination between the two subspecies to determine whether the acoustic differences we found here are important, and whether these differences constitute a reproductive barrier.

### Genetic divergence and song divergence

We used the divergence of cytochrome *b* sequences as a proxy for divergence between taxa [30] and assessed whether the time of the split between the taxa correlated with the magnitude of song divergence. This approach is similar to the one taken by [77] in different Amazonian forest systems, which found a correlation between overall song structure and genetic distance. Like our study, this approach assumes that genetic divergence in mtDNA is an accurate measure of divergence times, which has been quantified in other avian systems [78]. In our case, we found that the song differences did not correlate with mtDNA divergence, implying that these differences are either not related to geographic isolation during the Pleistocene or could not be captured via the coalesced timing of mtDNA (Fig 5 using Δ*p* as song divergence, Fig F in S1 File using PCA and PAM output as song divergence). Such timing could be confounded by processes such as mitochondrial introgression, which has occurred in one of the species pairs (*S*. *c*. *coronata* and *S*. *c*. *auduboni*; 54,56), though we attempted to control for this issue in this pair. It is also possible that divergence resulted in genome-wide effects, which might not have been adequately quantified by a single genetic marker like mtDNA. Future work reassessing the relationship between song divergence and whole genome data will be important to better identify the divergence patterns. We also cannot rule out the possibility that the timing of the isolation was not important, and these taxa would have diverged as long as they are isolated regardless of when. We also acknowledge that vocal traits could potentially evolve rather quickly; some species have been shown to change song characteristics over a short time frame: chestnut-sided warbler (*Setophaga pensylvanica*) showed new song elements within 19-year intervals [79]; song recognition in white-crowned sparrows (*Z*. *leucophrys*) for historical local songs is less in magnitude compared to current local songs and is proportional to how acoustically different the songs are, within about 20 years [80]. That said, we believe that among our study species, qualitative song characteristics have not changed since at least the 1950s, when the earliest recordings are available (e.g., a 1956 recording of *S*. *virens* is identical to a typical contemporary *S*. *virens* song; Macaulay Library Asset #ML67887).

### Caveats of this work

There are other aspects of vocalizations that we were not able to address in this study. First, we did not perform bioacoustic analysis for calls–another important vocalization signal for birds. Calls differ from songs in usage and function [81–83], and it was hypothesized that the evolutionary trajectories of the two types might be distinct because of these functional differences [84]. Moreover, calls tend to be more innate and would be more likely to be linked with genetic variation, the mechanism of which would also be different from how songs might correlate with genetic variation [84]. This further suggests that calls as a trait should be examined separately from song, which made it beyond the scope of our current study.

Moreover, we were not able to include many taxa that would have been appropriate for this comparative study. Though our chosen taxa are largely based on those chosen in [30] to leverage the divergence time estimated for the same pairs, species that were considered in that study, like *Passerella iliaca* subspecies and *Poecile rufescens* vs. *Poecile hudsonicus*, were not included here due to the lack of available DNA sequence for both counterparts. There were other pairs we could have examined, such as *Cyanocitta cristata* vs. *Cyanocitta stelleri*, or eastern vs. western *Sitta carolinensis*, but unfortunately could not include without significantly scaling up the scope of our study.

Additionally, suboscines were not included in our comparative analysis and therefore a diverse group of vocalizing species were excluded. This decision was based on our aim to investigate song divergence within the complex context of cultural evolution and its correlation with geographic differences, which are more relevant in species that learn songs compared to those that have innate songs. Although in this study we were not able to dissect whether the song divergence we observed was a consequence of cultural evolution or genetics, it is nevertheless crucial to quanitfy the magnitude of song divergence in oscines separately from suboscines. Because suboscines lack the exposure to errors and variation that can result in cultural mutation from the process of learning, we had good reason to hypothesize that there would be widespread geographic variation in oscines, but less so in suboscines [85–87] however see [88, 89]. The mechanism of song recognition is also different between oscines and suboscines, where suboscines were shown to recognize finer song differences to distinguish not only on a species level but also on an individual level (i.e., specific mate recognition, [90]). The species discrimination function of song recognition in suboscines is therefore different, in which songs can directly enable reproductive isolation and diversification [91]. Moreover, due to the difference in complexity of oscine vs. suboscine songs, it would be important to assess the overall trend of the song variation in each group independently rather than both groups in one analysis, especially in the comparative framework employed here.

## Conclusion

This work complements and extends previous comparative studies on the evolution of avian song evolution, song discrimination, and genetic divergence [92, 93]. Vocalization divergence in songbirds in North America has likely undergone both divergent and parallel evolution, which was greatly affected by isolation during glacial periods [84]. While our analysis of pairs shows this may not be directly related to time since their isolation, the consistent divergence between—though not necessarily within—each pair demonstrates these differences persist today. Future work incorporating genomic data will hopefully provide a more nuanced characterization of the size and diversity of refugial populations and, subsequently, how this may have influenced their cultural trajectory.

## Supporting information

S1 File Supporting information. Additional figures and tables: Fig A–Loading of PCA results for PC1 and PC2; Fig B–mtDNA cytb maximum-likelihood tree; Fig C–Clusters determined by partitioning around medoid (PAM) using R package ‘cluster’; Fig D–*Setophaga townsendi* regiolect; Fig E–*Icterus* and *Sturnella* additional analyses; Fig F–Spearman’s correlation between mtDNA divergence and song divergence quantified by PC1, PC2, PAM dimension 1, and PAM dimension 2; Table A–Summary of within-taxa regiolect detection in each counterpart; Table B–Dissimilarity matrices produced by PAM; Table C–The principal components 1–6 for each between-taxa comparison; Table D–The loading of principal components 1 & 2 for each variable measured for each between-taxa comparison.
(PDF)

## References

[1] Ebdon S, Laetsch DR, Dapporto L, Hayward A, Ritchie MG, Dincӑ V, et al. The Pleistocene species pump past its prime: Evidence from European butterfly sister species. Mol Ecol. 2021 Jul;30(14):3575–89. doi: 10.1111/mec.15981 33991396

[2] Garzón-Orduña IJ, Benetti-Longhini JE, Brower AVZ. Timing the diversification of the Amazonian biota: butterfly divergences are consistent with Pleistocene refugia. Riddle B, editor. J Biogeogr. 2014 Sep;41(9):1631–8.

[3] Lovette I. Glacial cycles and the tempo of avian speciation. Trends Ecol Evol. 2005 Feb;20(2):57–9. doi: 10.1016/j.tree.2004.11.011 16701342

[4] Levsen ND, Tiffin P, Olson MS. Pleistocene Speciation in the Genus Populus (Salicaceae). Syst Biol. 2012 May 1;61(3):401. doi: 10.1093/sysbio/syr120 22213709 PMC3529545

[5] Leavitt SD, Lumbsch HT, Stenroos S, Clair LLSt. Pleistocene Speciation in North American Lichenized Fungi and the Impact of Alternative Species Circumscriptions and Rates of Molecular Evolution on Divergence Estimates. Johnson N, editor. PLoS ONE. 2013 Dec 26;8(12):e85240. doi: 10.1371/journal.pone.0085240 24386465 PMC3873437

[6] Shafer ABA, Cullingham CI, Côté SD, Coltman DW. Of glaciers and refugia: A decade of study sheds new light on the phylogeography of northwestern North America. Mol Ecol. 2010 Nov;19(21):4589–621. doi: 10.1111/j.1365-294X.2010.04828.x 20849561

[7] Colbeck GJ, Gibbs HL, Marra PP, Hobson K, Webster MS. Phylogeography of a widespread North American migratory songbird (Setophaga ruticilla). J Hered. 2008 Sep 1;99(5):453–63. doi: 10.1093/jhered/esn025 18468988

[8] Hewitt GM. Genetic consequences of climatic oscillations in the Quaternary. Willis KJ, Bennett KD, Walker D, editors. Philos Trans R Soc Lond B Biol Sci. 2004 Feb 29;359(1442):183–95. 15101575 10.1098/rstb.2003.1388 PMC1693318

[9] Slabbekoorn H, Smith TB. Habitat-dependent song divergence in the little greenbul: an analysis of environmental selection pressures on acoustic signals. Evolution. 2002 Sep;56(9):1849–58. doi: 10.1111/j.0014-3820.2002.tb00199.x 12389730

[10] Edwards SV, Kingan SB, Calkins JD, Balakrishnan CN, Jennings WB, Swanson WJ, et al. Speciation in birds: Genes, geography, and sexual selection. Proc Natl Acad Sci U S A. 2005;102(SUPPL. 1):6550–7. doi: 10.1073/pnas.0501846102 15851678 PMC1131863

[11] Irwin DE. Culture in Songbirds and Its Contribution to the Evolution of New Species. In: Slingerland E, Collard M, editors. Creating Consilience: Integrating the Sciences and the Humanities [Internet]. Oxford University Press; 2011 [cited 2024 Mar 3]. p. 0. Available from: 10.1093/acprof:oso/9780199794393.003.0009

[12] Payne RB. 11. Song Traditions in Indigo Buntings: Origin, Improvisation, Dispersal, and Extinction in Cultural Evolution. In: Kroodsma DE, Miller EH, editors. Ecology and Evolution of Acoustic Communication in Birds [Internet]. Ithaca, NY: Cornell University Press; 2020 [cited 2024 Mar 3]. p. 198–220. Available from: https://www.degruyter.com/document/doi/10.7591/9781501736957-018/html

[13] Buainain N, Canton R, Zuquim G, Tuomisto H, Hrbek T, Sato H, et al. Paleoclimatic evolution as the main driver of current genomic diversity in the widespread and polymorphic Neotropical songbird *Arremon taciturnus*. Mol Ecol. 2020 Aug;29(15):2922–39. 32623766 10.1111/mec.15534

[14] Keighley MV, Heinsohn R, Langmore NE, Murphy SA, Peñalba JV. Genomic population structure aligns with vocal dialects in Palm Cockatoos (*Probosciger aterrimus*); evidence for refugial late-Quaternary distribution? Emu—Austral Ornithol. 2019 Jan 2;119(1):24–37.

[15] Pitocchelli J. Macrogeographic variation in the song of the Mourning warbler (Oporornis philadelphia). Can J Zool. 2011;89(11):1027–40.

[16] Toews DPL. From song dialects to speciation in white-crowned sparrows. Mol Ecol. 2017;26(11):2842–4. doi: 10.1111/mec.14104 28544663

[17] Kroodsma DE. Winter Wren Singing Behavior: A Pinnacle of Song Complexity. The Condor. 1980;82(4):357.

[18] Kroodsma DE. Two North American Song Populations of the Marsh Wren Reach Distributional Limits in the Central Great Plains. The Condor. 1989;91(2):332.

[19] Toews DPL, Irwin DE. Cryptic speciation in a Holarctic passerine revealed by genetic and bioacoustic analyses: SPECIATION IN WINTER WRENS. Mol Ecol. 2008 Jun;17(11):2691–705. 18444983 10.1111/j.1365-294X.2008.03769.x

[20] Wiley RH, Richards DG. Physical constraints on acoustic communication in the atmosphere: Implications for the evolution of animal vocalizations. Behav Ecol Sociobiol. 1978;3(1):69–94.

[21] Kroodsma DE, Canady RA. Differences in Repertoire Size, Singing Behavior, and Associated Neuroanatomy Among Marsh Wren Populations Have a Genetic Basis. The Auk. 1985 Jul 1;102(3):439–46.

[22] Baker MC, Spitler-Nabors KJ, Bradley DC. The response of female Mountain White-crowned Sparrows to songs from their natal dialect and an alien dialect. Behav Ecol Sociobiol. 1982 Jun;10(3):175–9.

[23] MacDougall-Shackleton EA, MacDougall-Shackleton SA. Cultural and genetic evolution in mountain white-crowned sparrows: Song dialects are associated with population structure. Evolution. 2001;55(12):2568–75. doi: 10.1111/j.0014-3820.2001.tb00769.x 11831670

[24] Soha JA. Genetic analysis of song dialect populations in Puget Sound white-crowned sparrows. Behav Ecol. 2004 Jul 1;15(4):636–46.

[25] Lipshutz SE, Overcast IA, Hickerson MJ, Brumfield RT, Derryberry EP. Behavioural response to song and genetic divergence in two subspecies of white-crowned sparrows (Zonotrichia leucophrys). Mol Ecol. 2017;26(11):3011–27. doi: 10.1111/mec.14002 28036146

[26] Martens J. Vocalizations and Speciation of Palearctic Birds. In: Kroodsma DE, Miller EH, editors. Ecology and Evolution of Acoustic Communication in Birds. Ithaca, NY: Cornell University Press; 1996. p. 221–40.

[27] Delmore KE, Kenyon HL, Germain RR, Irwin DE. Phenotypic divergence during speciation is inversely associated with differences in seasonal migration. Proc R Soc B Biol Sci. 2015 Nov 22;282(1819):20151921. doi: 10.1098/rspb.2015.1921 26559951 PMC4685813

[28] Martin PR, Montgomerie R, Lougheed SC. Rapid sympatry explains greater color pattern divergence in high latitude birds. Evolution. 2010 Feb;64(2):336–47. doi: 10.1111/j.1558-5646.2009.00831.x 19744123

[29] Martin PR, McKay JK. Latitudinal variation in genetic divergence of populations and the potential for future speciation. Evolution. 2004 May;58(5):938–45. doi: 10.1111/j.0014-3820.2004.tb00428.x 15212375

[30] Weir JT, Schluter D. Ice sheets promote speciation in boreal birds. Proc R Soc B Biol Sci. 2004 Sep 22;271(1551):1881–7. doi: 10.1098/rspb.2004.2803 15347509 PMC1691815

[31] Rubinoff D, Holland BS. Between Two Extremes: Mitochondrial DNA is neither the Panacea nor the Nemesis of Phylogenetic and Taxonomic Inference. Savolainen V, editor. Syst Biol. 2005 Dec 1;54(6):952–61. 16385775 10.1080/10635150500234674

[32] Rheindt FE, Edwards SV. Genetic Introgression: An Integral but neglected component of speciation in birds. The Auk. 2011 Oct;128(4):620–32.

[33] Barker FK, Vandergon AJ, Lanyon SM. Assessment of species limits among yellow-breasted meadowlarks (sturnella spp.) using mitochondrial and sex-linked markers. Auk. 2008;125(4):869–79.

[34] Cicero C, Johnson NK. Molecular phylogeny and ecological diversification in a clade of New World songbirds (genus Vireo). Mol Ecol. 1998 Oct;7(10):1359–70. doi: 10.1046/j.1365-294x.1998.00483.x 9787446

[35] Jacobsen F, Friedman NR, Omland KE. Congruence between nuclear and mitochondrial DNA: Combination of multiple nuclear introns resolves a well-supported phylogeny of New World orioles (Icterus). Mol Phylogenet Evol. 2010 Jul 1;56(1):419–27. doi: 10.1016/j.ympev.2010.03.035 20363347

[36] Lovette IJ, Pérez-Emán JL, Sullivan JP, Banks RC, Fiorentino I, Córdoba-Córdoba S, et al. A comprehensive multilocus phylogeny for the wood-warblers and a revised classification of the Parulidae (Aves). Mol Phylogenet Evol. 2010;57(2):753–70. doi: 10.1016/j.ympev.2010.07.018 20696258

[37] Slager DL, Battey CJ, Bryson RW, Voelker G, Klicka J. A multilocus phylogeny of a major New World avian radiation: The Vireonidae. Mol Phylogenet Evol. 2014 Nov 1;80(1):95–104. 25109651 10.1016/j.ympev.2014.07.021

[38] Morse DH, Poole AF. Black-throated Green Warbler (Setophaga virens). In: Billerman SM, Keeney BK, Rodewald PG, Schulenberg TS, editors. Birds of the World [Internet]. Cornell Lab of Ornithology; 2020 [cited 2024 Mar 4]. Available from: https://birdsoftheworld.org/bow/species/btnwar/1.0/introduction

[39] Wright AL, Hayward GD, Matsuoka SM, Hayward PH. Townsend’s Warbler (Setophaga townsendi). In: Billerman SM, Keeney BK, Rodewald PG, Schulenberg TS, editors. Birds of the World [Internet]. Cornell Lab of Ornithology; 2020 [cited 2024 Mar 4]. Available from: https://birdsoftheworld.org/bow/species/towwar/1.0/introduction

[40] Kimura M, Clegg SM, Lovette IJ, Holder KR, Girman DJ, Mila B, et al. Phylogeographical approaches to assessing demographic connectivity between breeding and overwintering regions in a Nearctic-Neotropical warbler (Wilsonia pusilla). Mol Ecol. 2002 Sep;11(9):1605–16. doi: 10.1046/j.1365-294x.2002.01551.x 12207712

[41] Esri. "Outline" [basemap]. Scale Not Given. "Outline". Apr 25, 2024. https://pennstate.maps.arcgis.com/home/item.html?id=ba99a4a4f5ce48debbeca6713e051f1e (May 15, 2024).

[42] Kenyon HL, Alcaide M, Toews DPL, Irwin DE. Cultural isolation is greater than genetic isolation across an avian hybrid zone. J Evol Biol. 2017 Jan;30(1):81–95. doi: 10.1111/jeb.12989 27732753

[43] The Cornell Lab of Ornithology. Raven Pro: Interactive Sound Analysis Software [Internet]. Ithaca, NY: K. Lisa Yang Center for Conservation Bioacoustics at the Cornell Lab of Ornithology; 2022. Available from: https://ravensoundsoftware.com/

[44] K. Lisa Yang Center for Conservation Bioacoustics at the Cornell Lab of Ornithology. Raven Sound Analysis. 2022. Robust Signal Measurements. Available from: https://ravensoundsoftware.com/knowledge-base/robust-signal-measurements/

[45] Maechler M, Rousseeuw P, Struyf A, Hubert M, Hornik K. cluster: Cluster Analysis Basics and Extensions [Internet]. 2022. Available from: https://CRAN.R-project.org/package=cluster

[46] Gower JC. A General Coefficient of Similarity and Some of Its Properties. Biometrics. 1971 Dec;27(4):857.

[47] Hothorn T, Hornik K, Wiel MAVD, Zeileis A. Implementing a Class of Permutation Tests: The coin Package. J Stat Soft. 2008 Nov;28(8):1–23:v028i08

[48] Chavent M, Kuentz-Simonet V, Labenne A, Saracco J. Multivariate Analysis of Mixed Data: The R Package PCAmixdata [Internet]. arXiv; 2022 [cited 2022 Dec 26]. Available from: http://arxiv.org/abs/1411.4911

[49] BirdLife International and Handbook of the Birds of the World. Bird species distribution maps of the world [Internet]. 2021. Available from: http://datazone.birdlife.org/species/requestdis

[50] Safran R, Flaxman S, Kopp M, Irwin DE, Briggs D, Evans MR, et al. A robust new metric of phenotypic distance to estimate and compare multiple trait differences among populations. Curr Zool. 2012 Jun 1;58(3):426–39.

[51] Tamura K, Stecher G, Kumar S. MEGA11: Molecular Evolutionary Genetics Analysis Version 11. Mol Biol Evol. 2021 Jul 1;38(7):3022–7. doi: 10.1093/molbev/msab120 33892491 PMC8233496

[52] Mello B. Estimating TimeTrees with MEGA and the TimeTree Resource. Dudley J, editor. Molecular Biology and Evolution. 2018 Sep 1;35(9):2334–42. 29931306 10.1093/molbev/msy133

[53] Runfola D, Anderson A, Baier H, Crittenden M, Dowker E, Fuhrig S, et al. geoBoundaries: A global database of political administrative boundaries. Tang W, editor. PLoS ONE. 2020 Apr 24;15(4):e0231866. 10.1371/journal.pone.0231866 PMC7182183 32330167

[54] Derryberry EP. Ecology Shapes Birdsong Evolution: Variation in Morphology and Habitat Explains Variation in White‐Crowned Sparrow Song. Am Nat. 2009 Jul;174(1):24–33. doi: 10.1086/599298 19441960

[55] Slabbekoorn H. Songs of the city: noise-dependent spectral plasticity in the acoustic phenotype of urban birds. Anim Behav. 2013 May;85(5):1089–99.

[56] Phillips JN, Rochefort C, Lipshutz S, Derryberry GE, Luther D, Derryberry EP. Increased attenuation and reverberation are associated with lower maximum frequencies and narrow bandwidth of bird songs in cities. J Ornithol. 2020 Apr;161(2):593–608.

[57] Ore MJ, Wang S, Irwin DE. Gradual transitions in genetics and songs between coastal and inland populations of *Setophaga townsendi*. Ornithology. 2023 May 8;140(2):ukac060.

[58] Beam JK, Funk ER, Taylor SA. Genomic and acoustic differences separate Lilian’s Meadowlark (*Sturnella magna lilianae*) from Eastern (*S*. *magna*) and Western (*S*. *neglecta*) meadowlarks. Ornithology. 2021 May 5;138(2):ukab004.

[59] Irwin DE, Brelsford A, Toews DPL, MacDonald C, Phinney M. Extensive hybridization in a contact zone between MacGillivray’s warblers *Oporornis tolmiei* and mourning warblers *O*. *philadelphia* detected using molecular and morphological analyses. J Avian Biol. 2009 Sep;40(5):539–52.

[60] Kenyon HL, Toews DPL, Irwin DE. Can Song Discriminate between MacGillivray’s and Mourning Warblers in a Narrow Hybrid Zone? The Condor. 2011 Aug;113(3):655–63.

[61] Brelsford A. Hybridization and speciation in the Yellow-rumped Warbler complex. 2010 [cited 2022 Dec 23]; Available from: https://doi.library.ubc.ca/ doi: 10.14288/1.0071052

[62] Carling MD, Serene LG, Lovette IJ. Using Historical DNA to Characterize Hybridization Between Baltimore Orioles (*Icterus galbula*) and Bullock’s Orioles (*I*. *bullockii*). The Auk. 2011 Jan;128(1):61–8.

[63] Toews DPL, Brelsford A, Grossen C, Milá B, Irwin DE. Genomic variation across the Yellow-rumped Warbler species complex. The Auk. 2016 Oct;133(4):698–717.

[64] Walsh J, Billerman SM, Rohwer VG, Butcher BG, Lovette IJ. Genomic and plumage variation across the controversial Baltimore and Bullock’s oriole hybrid zone. Auk. 2021 Feb 1;137(4).

[65] Walsh J, Billerman SM, Butcher BG, Rohwer VG, Toews DPL, Vila-Coury V, et al. A complex genomic architecture underlies reproductive isolation in a North American oriole hybrid zone. Commun Biol. 2023 Feb 7;6(1):154. doi: 10.1038/s42003-023-04532-8 36747071 PMC9902562

[66] Abzhanov A, Kuo WP, Hartmann C, Grant BR, Grant PR, Tabin CJ. The calmodulin pathway and evolution of elongated beak morphology in Darwin’s finches. Nature. 2006 Aug;442(7102):563–7. doi: 10.1038/nature04843 16885984

[67] Cahill JA, Armstrong J, Deran A, Khoury CJ, Paten B, Haussler D, et al. Positive selection in noncoding genomic regions of vocal learning birds is associated with genes implicated in vocal learning and speech functions in humans. Genome Res. 2021 Nov;31(11):2035–49. doi: 10.1101/gr.275989.121 34667117 PMC8559704

[68] Irwin DE, Irwin JH, Smith TB. Genetic variation and seasonal migratory connectivity in Wilson’s warblers (Wilsonia pusilla): species-level differences in nuclear DNA between western and eastern populations: MIGRATORY CONNECTIVITY AND GENETIC DIVERGENCE. Mol Ecol. 2011 Aug;20(15):3102–15. 21689190 10.1111/j.1365-294X.2011.05159.x

[69] Pyle P, Howell SNG. Identification guide to North American birds. Bolinas, CA: Slate Creek Press; 1997. 1 p.

[70] Ruegg KC, Anderson EC, Paxton KL, Apkenas V, Lao S, Siegel RB, et al. Mapping migration in a songbird using high-resolution genetic markers. Mol Ecol. 2014 Dec;23(23):5726–39. doi: 10.1111/mec.12977 25346105

[71] Goguen C, Curson DR. Cassin’s Vireo (Vireo cassinii). In: Billerman SM, Keeney BK, Rodewald PG, Schulenberg TS, editors. Birds of the World [Internet]. Cornell Lab of Ornithology; 2020 [cited 2022 Dec 26]. Available from: https://birdsoftheworld.org/bow/species/casvir/1.0/introduction

[72] Murray BW, McGillivray WB, Barlow JC, Beech RN, Strobeck C. The Use of Cytochrome b Sequence Variation in Estimation of Phylogeny in the Vireonidae. The Condor. 1994 Nov;96(4):1037–54.

[73] Lowther PE, Williams JMcl. Nashville Warbler (Leiothlypis ruficapilla). In: Billerman SM, Keeney BK, Rodewald PG, Schulenberg TS, editors. Birds of the World [Internet]. Cornell Lab of Ornithology; 2020 [cited 2024 Feb 8]. Available from: https://birdsoftheworld.org/bow/species/naswar/1.0/introduction

[74] Simpson RK, Wilson DR, Mistakidis AF, Mennill DJ, Doucet SM. Sympatry drives colour and song evolution in wood-warblers (Parulidae). Proc R Soc B Biol Sci. 2021 Jan 13;288(1942):20202804. doi: 10.1098/rspb.2020.2804 33434456 PMC7892414

[75] Ey E, Fischer J. The “Acoustic Adaptation Hypothesis”—a review of the evidence from birds, anurans and mammals. Bioacoustics. 2009 Jan;19(1–2):21–48.

[76] Lovette IJ, Clegg SM, Smith TB. Limited Utility of mtDNA Markers for Determining Connectivity among Breeding and Overwintering Locations in Three Neotropical Migrant Birds. Conserv Biol. 2004;18(1):156–66.

[77] Tobias JA, Aben J, Brumfield RT, Derryberry EP, Halfwerk W, Slabbekoorn H, et al. SONG DIVERGENCE BY SENSORY DRIVE IN AMAZONIAN BIRDS: SENSORY DRIVE IN BIRDSONG EVOLUTION. Evolution. 2010 Aug 19;no-no. 10.1111/j.1558-5646.2010.01067.x 20561048

[78] Weir JT, Schluter D. Calibrating the avian molecular clock. Mol Ecol. 2008 May;17(10):2321–8. doi: 10.1111/j.1365-294X.2008.03742.x 18422932

[79] Byers BE, Belinsky KL, Bentley RA. Independent Cultural Evolution of Two Song Traditions in the Chestnut‐Sided Warbler. Am Nat. 2010 Oct;176(4):476–89. doi: 10.1086/656268 20712515

[80] Derryberry EP. Male response to historical and geographical variation in bird song. Biol Lett. 2011 Feb 23;7(1):57–9. doi: 10.1098/rsbl.2010.0519 20685696 PMC3030877

[81] den Hartog PM, Slabbekoorn H, ten Cate C. Male territorial vocalizations and responses are decoupled in an avian hybrid zone. Philos Trans R Soc B Biol Sci. 2008 Sep 12;363(1505):2879–89. 10.1098/rstb.2008.0046 PMC2606739 18508751

[82] Marler P. Bird calls: a cornucopia for communication. In: Nature’s Music [Internet]. Elsevier; 2004 [cited 2022 Sep 13]. p. 132–77. Available from: https://linkinghub.elsevier.com/retrieve/pii/B9780124730700500086

[83] Wheatcroft D, Price TD. Learning and signal copying facilitate communication among bird species. Proc R Soc B Biol Sci. 2013 Apr 22;280(1757):20123070. doi: 10.1098/rspb.2012.3070 23446529 PMC3619484

[84] Irwin DE, Thimgan MP, Irwin JH. Call divergence is correlated with geographic and genetic distance in greenish warblers (Phylloscopus trochiloides): A strong role for stochasticity in signal evolution? J Evol Biol. 2008;21(2):435–48. doi: 10.1111/j.1420-9101.2007.01499.x 18205774

[85] Acero-Murcia AC, Raposo do Amaral F, de Barros FC, da Silva Ribeiro T, Miyaki CY, Maldonado-Coelho M. Ecological and evolutionary drivers of geographic variation in songs of a Neotropical suboscine bird: The Drab-breasted Bamboo Tyrant (*Hemitriccus diops*, Rhynchocyclidae). Ornithology. 2021 May 5;138(2):ukab003.

[86] Foote JR, Palazzi E, Mennill DJ. Songs of the Eastern Phoebe, a suboscine songbird, are individually distinctive but do not vary geographically. Bioacoustics. 2013 Jun;22(2):137–51.

[87] Lein MR. Song Variation In Buff-Breasted Flycatchers (Empidonax fulvifrons). Wilson J Ornithol. 2008 Jun;120(2):256–67.

[88] Capelli D, Batalha-Filho H, Japyassú HF. Song variation in the Caatinga suboscine Silvery-cheeked Antshrike (Sakesphorus cristatus) suggests latitude and São Francisco River as drivers of geographic variation. J Ornithol. 2020 Jul;161(3):873–84.

[89] Isler ML, Isler PR, Brumfield RT. Clinal Variation in Vocalizations of an Antbird (Thamnophilidae) and Implications for Defining Species Limits. Zink RM, editor. The Auk. 2005 Apr 1;122(2):433–44.

[90] Seddon N, Tobias JA. Character displacement from the receiver’s perspective: species and mate recognition despite convergent signals in suboscine birds. Proc R Soc B Biol Sci. 2010 Aug 22;277(1693):2475–83. doi: 10.1098/rspb.2010.0210 20375056 PMC2894922

[91] Seddon N, Merrill RM, Tobias JA. Sexually Selected Traits Predict Patterns of Species Richness in a Diverse Clade of Suboscine Birds. Am Nat. 2008 May;171(5):620–31. doi: 10.1086/587071 18419570

[92] Freeman BG, Montgomery GA, Heavyside J, Moncrieff AE, Johnson O, Winger BM. On the predictability of phenotypic divergence in geographic isolation. Evolution. 2023 Jan 23;77(1):26–35. doi: 10.1093/evolut/qpac040 36622803

[93] Weir JT, Price TD. Song playbacks demonstrate slower evolution of song discrimination in birds from Amazonia than from temperate North America. PLoS Biol. 2019;17(10):1–19. doi: 10.1371/journal.pbio.3000478 31639139 PMC6804960

