# Supplementary material for: Comparative bioacoustics of multiple eastern versus western songbird pairs in North America reveals a gradient of song divergence
Source: PLoS One. 2024 Dec 26;19(12):e0312706. doi: 10.1371/journal.pone.0312706 (PMC11670943; doi:10.1371/journal.pone.0312706)
Supplement: S1 File — Additional figures and tables: Fig A–Loading of PCA results for PC1 and PC2; Fig B–mtDNA cytb maximum-likelihood tree; Fig C–Clusters determined by partitioning around medoid (PAM) using R package ‘cluster’; Fig D–Setophaga townsendi regiolect; Fig E–Icterus and Sturnella additional analyses; Fig F–Spearman’s correlation between mtDNA divergence and song divergence quantified by PC1, PC2, PAM dimension 1, and PAM dimension 2; Table A–Summary of within-taxa regiolect detection in each counterpart; Table B–Dissimilarity matrices produced by PAM; Table C–The principal components 1–6 for each between-taxa comparison; Table D–The loading of principal components 1 & 2 for each variable measured for each between-taxa comparison. (PDF) [file pone.0312706.s001.pdf]

Supplemental Material

Fig A. Loading of PCA results for PC1 and PC2. All measurements mentioned in Table 1 is presented for each pair.

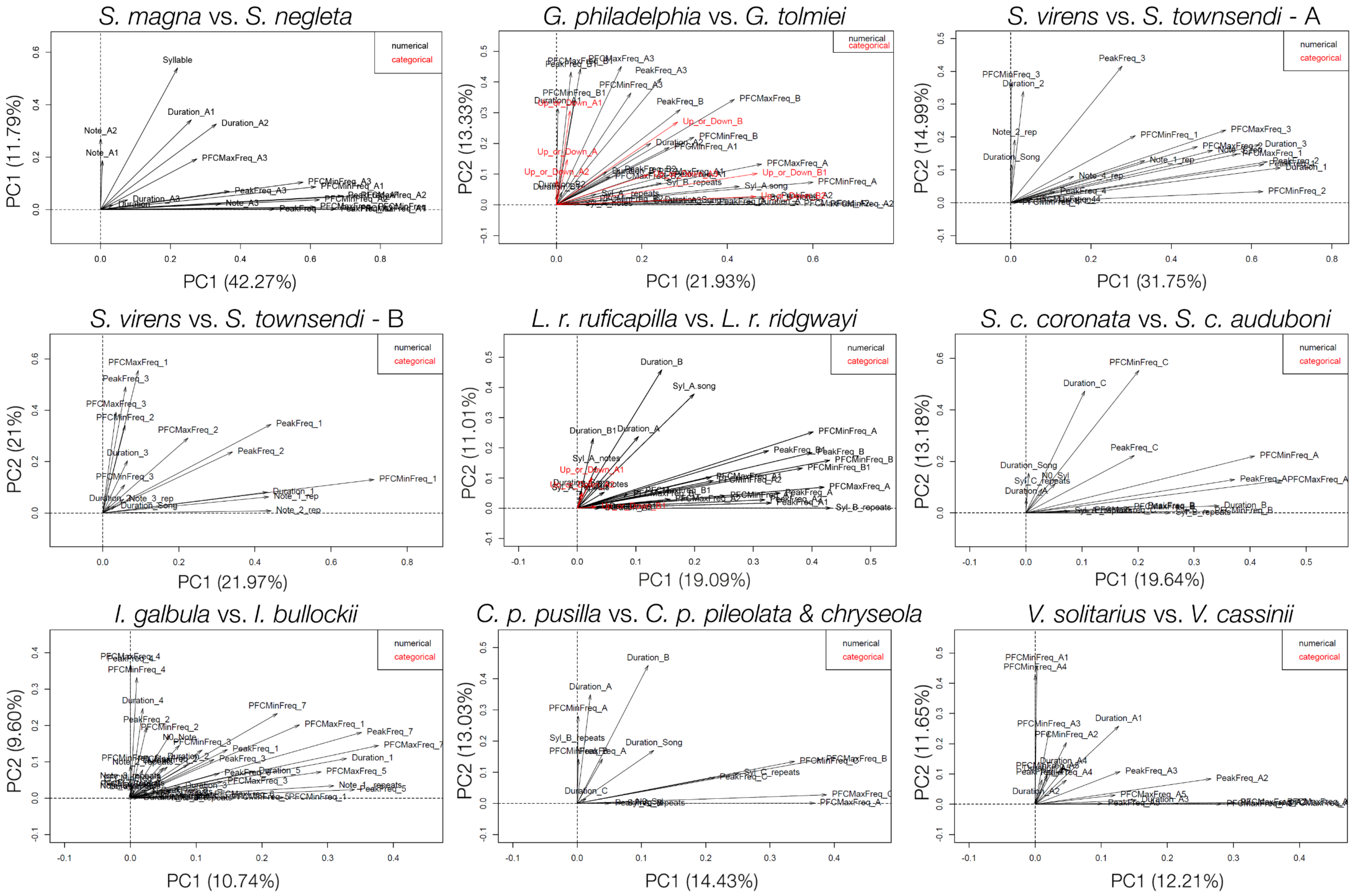

Fig B. mtDNA *cytb* maximum-likelihood tree.

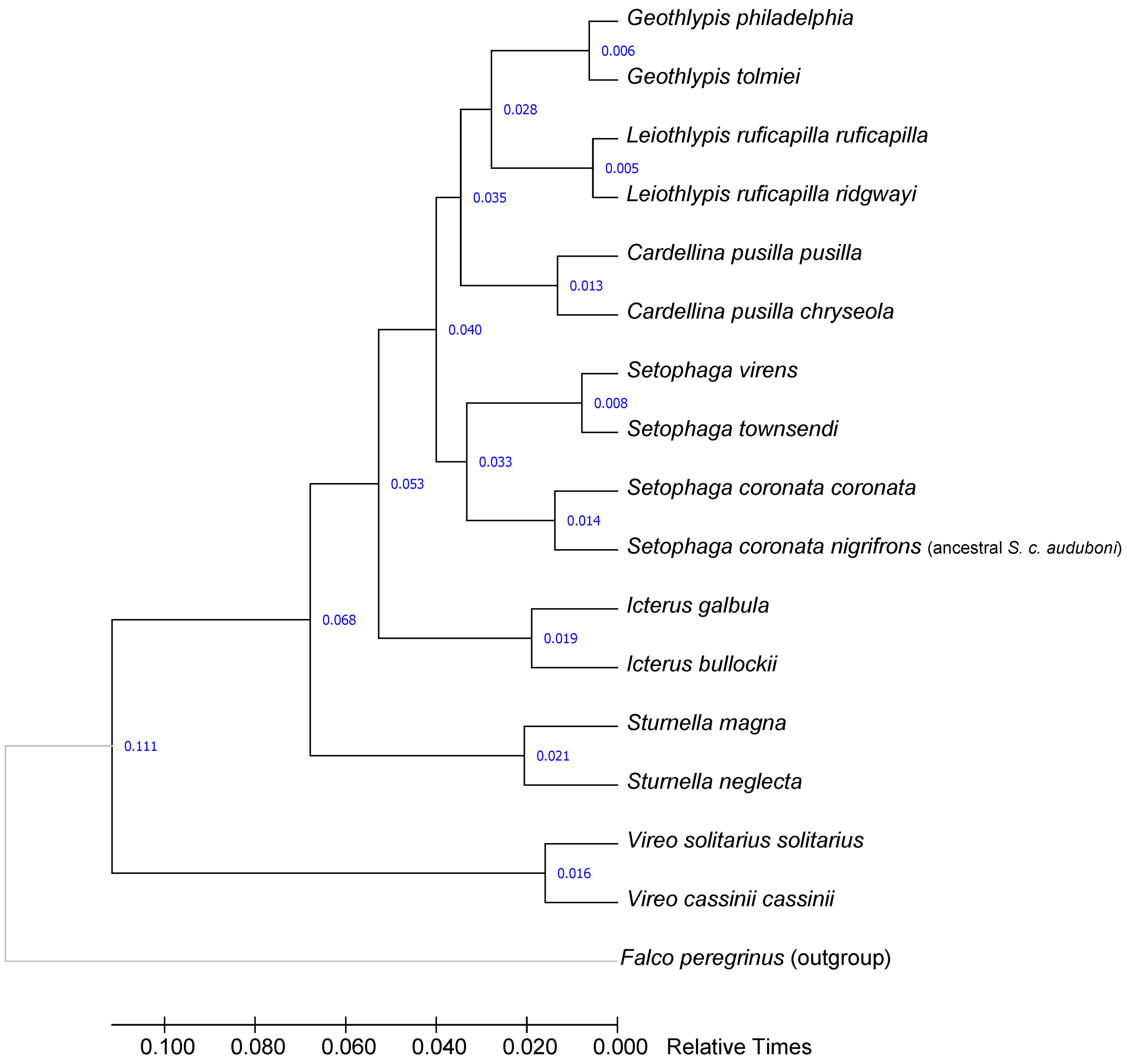

Fig C. Clusters determined by partitioning around medoid (PAM) using R package ‘cluster’. The number of best-fit cluster was determined by silhouette width, native to the R package.

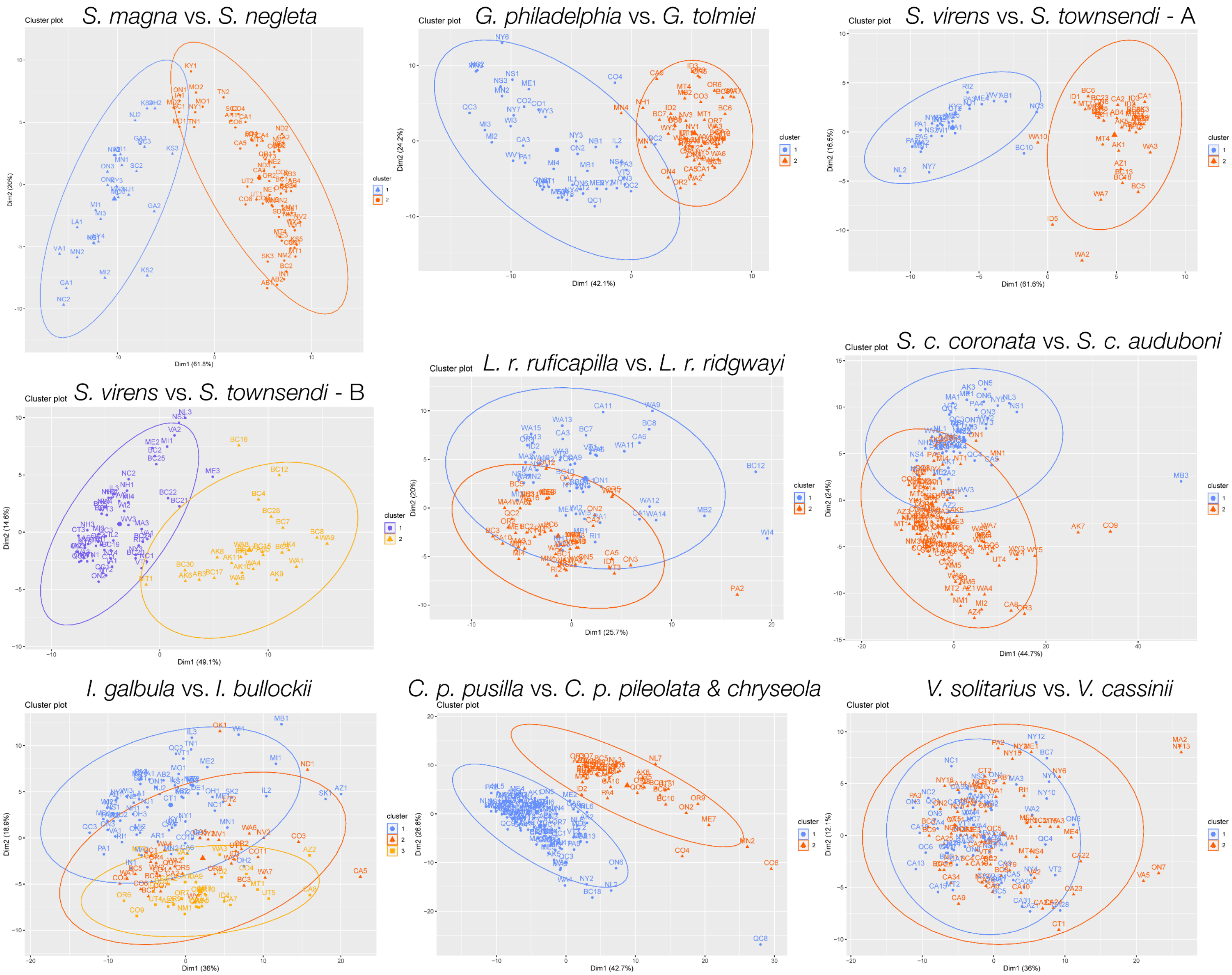

Fig D. *Setophaga townsendi* regiolect. (a) PCA of type B song from the initial set of *S. townsendi* (n=32); (b) PCA of type B song with additional samples from the British Columbia mainland area (n=42), which showed the same pattern as (a), only that the loading of PC1—which contains the highest loading variable, maximum frequency of syllable C—is higher. The remaining panels show the result of the analyses including additional data (n=42): (c) Clusters determined by PAM; (d) t-test of the highest loading variable maximum frequency of syllable C; t_{23} = -5.14, P=3.11x10^{-5}; (e) map of clusters in (c), showing that the groupings are concordant with geographic variation.

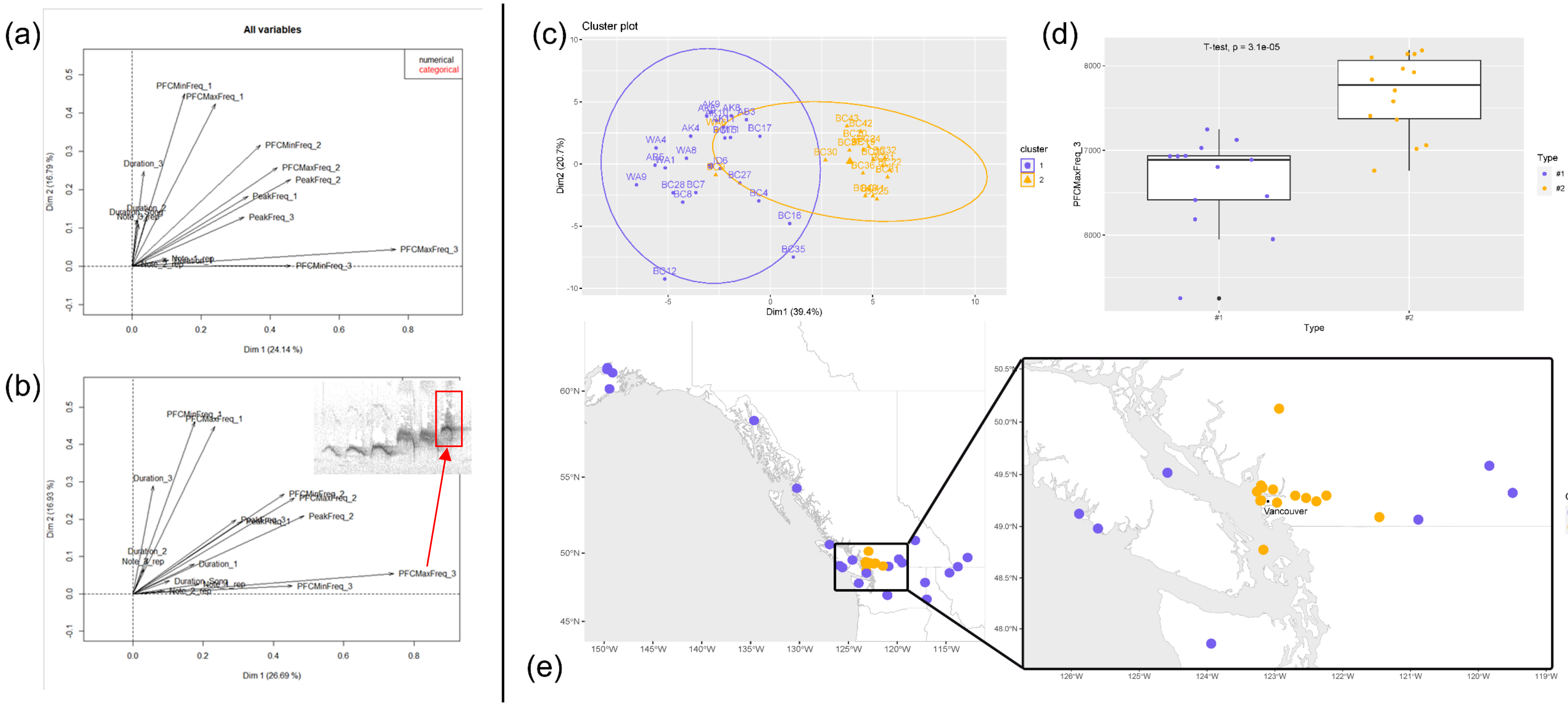

Fig E. *Icterus* dataset and *Sturnella* dataset contains many samples identified as either “female” or “unknown” songs, due to the prevalence of female songs in *Icterus* and the difficulty of visually identifying sex in *Sturnella*. We performed PCA to include sex to the datasets to identify if this potential structure in the dataset might affect our findings. For both *S. magna* vs. *S. negleta* (panel a) and *I. galbula* vs. *I. bullockii* (panel b), we found that sex does not contribute to the differentiation between songs. Moreover, the proportion of “male”, “female” and “unknown” songs are not significantly different between acoustic-based clusters generated by PAM presented in the main text. In other words, female song did not differ from male song, and the sex of “unknown” songs cannot be identified as either sex.

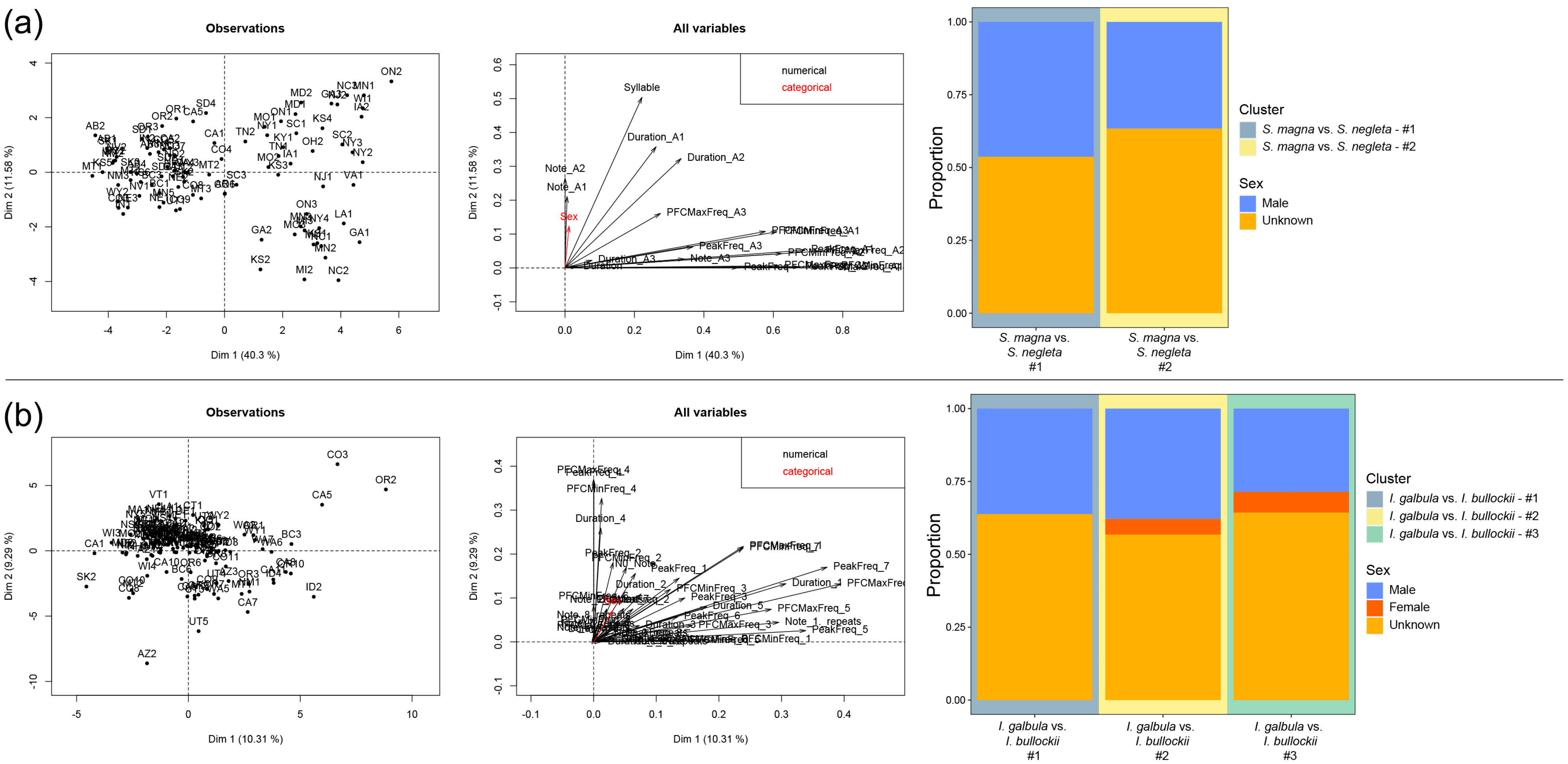

Fig F. Spearman's correlation between mtDNA divergence and song divergence quantified by PC1, PC2, PAM dimension 1, and PAM dimension 2. All tests showed no correlation between mtDNA divergence and song divergence.

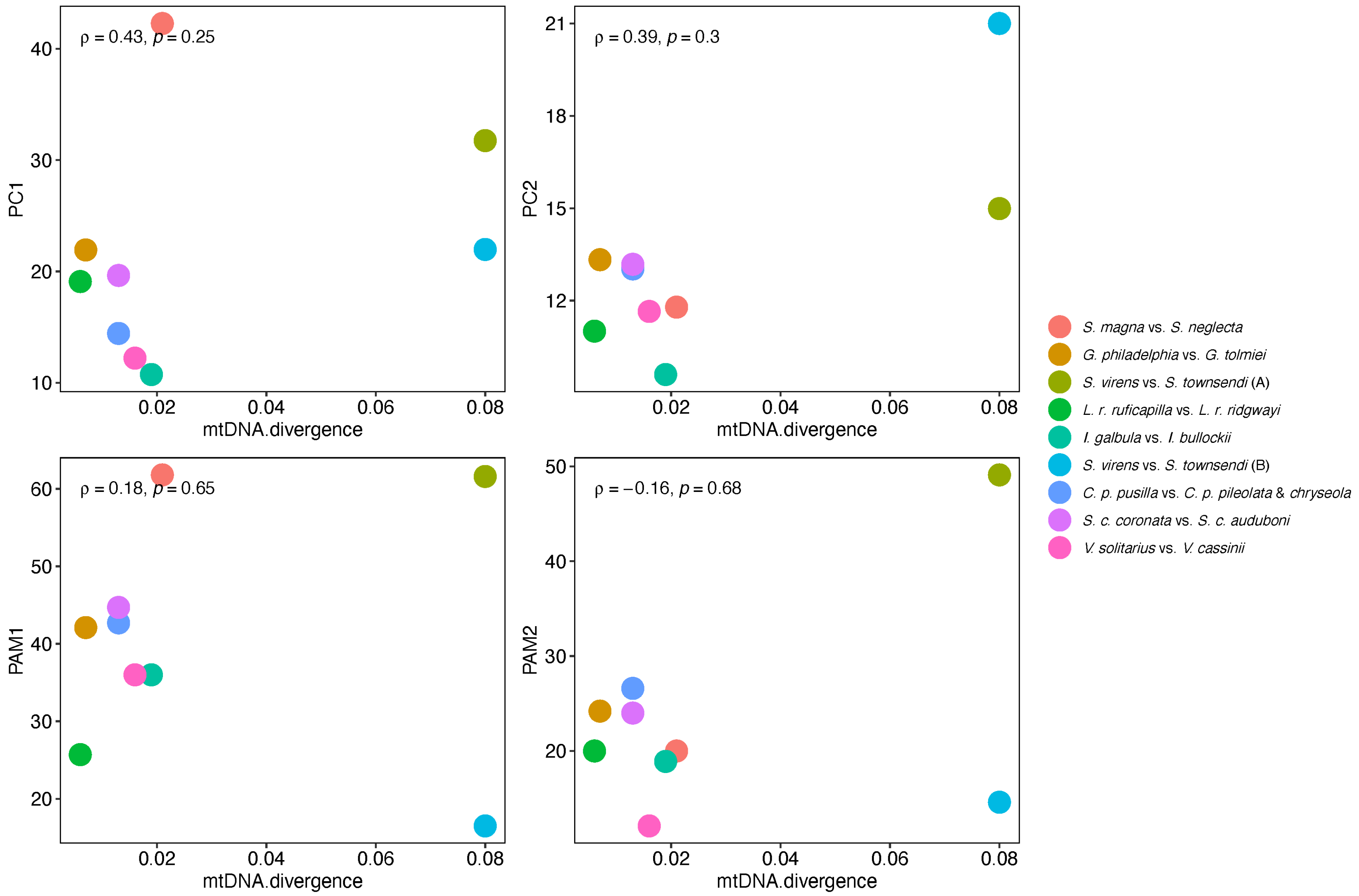

Table A. Summary of within-taxa regiolect detection in each counterpart.

	PCA (PC1, PC2)	PAM	Geographic variation
S. magna	24.02%, 15.17%	2	None
S. neglecta	22.4%, 15.4%	2	None
G. philadelphia	21.17%, 14.16%	3	None
G. tolmiei	18.47%, 11.86%	2	Potential coastal vs. inland regiolect
S. virens	A: 26.36%, 23.09%<br>B: 47.65%, 14.75%	A: 3<br>B: 2	None
S. townsendi	A: 34.32%, 14.15%<br>B: 24.14%, 16.79%	A: 2<br>B: 2	A: None<br>B: Clear regiolect in Vancouver
L. r. ruficapilla	21.51%, 13.22%	3	None
L.r. ridgwayi	18.77%, 12.74%	2	None
S. c. coronata	19.98%, 16.21%	2	One main song & one variant
S. c. auduboni	21.25%, 14.49%	1	None
I. galbula	13.07%, 11.62%	2	None
I. bullockii	11.87%, 9.734%	2	Two equally prominent variations; not geographically related
C. p. pusilla	17.39%, 15%	2	One main song & one variant; not geographically related
C. p. pileolata & *chryseola*	15.55%, 13.68%	2	Potential coastal vs. inland regiolect; one group is close to *C. p. pusilla*
V. solitarius	12.18%, 11.25%	2	None
V. cassinii	15.27%, 11.02%	2	None

Table B. Dissimilarity matrices produced by PAM is a metric of how different (dissimilar) acoustics characteristics are. The *p*-values below are pairwise comparisons between the means of dissimilarity scores, which assess whether the dissimilarity between taxa is larger than the dissimilarity within taxa.

	Group 1 – Group 2	Adjusted *p*-value (permutation test)
	S. magna – *S. neglecta*	0 ****
	S. magna x *S. neglecta* – *S. magna*	5.99×10^{-151} ****
	S. magna x *S. neglecta* – *S. neglecta*	0 ****
	G. philadelphia – *G. tolmiei*	8.15×10^{-3} ****
	G. philadelphia x *G. tolmiei* – *G . philadelphia*	1.73×10^{-106} ****
	G. philadelphia x *G. tolmiei* – *G. tolmiei*	0 ****
	S. virens (A) – *S. townsendi* (A)	7.11×10^{-30} ****
	S. virens (A) x *S. townsendi* (A) – *S. virens* (A)	5.34×10^{-158} ****
	S. virens (A) x *S. townsendi* (A) – *S. townsendi* (A)	0 ****

	S. virens (B) – *S. townsendi* (B)	$7x10^{-73}$ ****
	S. virens (B) x *S. townsendi* (B) – *S. virens* (B)	$7.76x10^{-248}$ ****
	S. virens (B) x *S. townsendi* (B) – *S. townsendi* (B)	$3.11x10^{-15}$ ****
	L. r. ruficapilla – *L.r. ridgwayi*	$1.2x10^{-8}$ ****
	L. r. ruficapilla x *L.r. ridgwayi* – *L. r. ruficapilla*	$3.32x10^{-44}$ ****
	L. r. ruficapilla x *L.r. ridgwayi* – *L.r. ridgwayi*	$4.99x10^{-13}$ ****
	S. c. coronata – *S. c. auduboni*	$7.99x10^{-3}$ ****
	S. c. coronata x *S. c. auduboni* – *S. c. coronata*	$7.18x10^{-38}$ ****
	S. c. coronata x *S. c. auduboni* – *S. c. auduboni*	0 ****
	I. galbula – *I. bullockii*	$4.06x10^{-42}$ ****
	I. galbula x *I. bullockii* – *I. galbula*	$4.59x10^{-179}$ ****
	I. galbula x *I. bullockii* – *I. bullockii*	0 ****
	C. p. pusilla – *C. p. pileolata* & *chryseola*	$7.99x10^{-3}$ ****
	C. p. pusilla x *C. p. pileolata* & *chryseola* – *C. p. pusilla*	$7.18x10^{-38}$ ****
	C. p. pusilla x *C. p. pileolata* & *chryseola* – *C. p. pileolata* & *chryseola*	0 ****
	V. solitarius – *V. cassinii*	0 ****
	V. solitarius x *V. cassinii* – *V. solitarius*	0.3093 (ns)
	V. solitarius x *V. cassinii* – *V. cassinii*	0 ****

Table C. The principal components 1-6 for each between-taxa comparison.

	**PC1 (%)**	**PC2 (%)**	**PC3 (%)**	**PC4(%)**	**PC5 (%)**	**PC6 (%)**
S. magna vs. *neglecta*	42.27	11.79	10.35	6.54	5.42	4.47
G. philadelphia vs. *tolmiei*	21.93	13.33	8.18	6.58	5.80	4.99
S. virens vs. *townsendi*	A: 31.75<br>B: 21.97	A: 14.99<br>B: 21	A: 11.26<br>B: 14.42	A: 6.39<br>B: 9.48	A: 6.16<br>B: 7.57	A: 5.12<br>B: 6.64
L. r. ruficapilla vs. *ridgwayi*	19.09	11.01	10.70	8.20	7.89	5.82
S. c. coronata vs. *auduboni*	19.64	13.18	11.06	10.11	7.64	6.38
I. galbula vs. *bullockii*	10.74<br>Log: 18.4	9.602<br>Log: 14.5	7.78<br>Log: 10.8	7.34<br>Log: 8.3	5.97<br>Log: 7.4	5.70<br>Log: 6.8
C. p. pusilla vs. *pileolata* & *chryseola*	14.43	13.03	10.71	9.73	8.48	7.24
V. solitarius vs. *cassinii*	12.21	11.65	8.03	7.40	6.94	6.34

Table D. The loading of principal components 1 & 2 for each variable measured for each between-taxa comparison. The cells are colored according to their values from low (yellow) to high (purple).

Lowest loading — Highest loading

	S. magna vs. *neglecta*		*G. philadelphia* vs. *tolmiei*		*S. virens* vs. *townsendi* (A)		*S. virens* vs. *townsendi* (B)		*I. galbula* vs. *bullockii*		*C. p. pusilla* vs. *pileolata* & *chryseola*		*L. r. ruficapilla* vs. *ridgwayi*		*S. c. coronata* vs. *auduboni*		*V. solitarius* vs. *cassinii*	
Variables	PC1	PC2	PC1	PC2	PC1	PC2	PC1	PC2	PC1	PC2	PC1	PC2	PC1	PC2	PC1	PC2	PC1	PC2
**1.** Duration (s)	0.02	0	0.24	0.02	0	0.12	0.03	0.03	0.01	0	0.12	0.17	0.01	0.06	0.01	0.15	--	--
**2.** Peak frequency (Hz)	0.49	0.01	--	--	--	--	--	--	--	--	--	--	--	--	--	--	--	--
**3.** Minimum frequency (Hz)	0.77	0	--	--	--	--	--	--	--	--	--	--	--	--	--	--	--	--
**4.** Maximum frequency (Hz)	0.59	0	--	--	--	--	--	--	--	--	--	--	--	--	--	--	--	--
**7.** Number of syllables	0.19	0.54	--	--	--	--	--	--	--	--	0.08	0	--	--	--	--	--	--
**9.** Proportion of the song that is syllable A (%)	--	--	0.43	0.06	--	--	--	--	--	--	--	--	0.2	0.38	--	--	--	--
**1a.** Duration of syllable A (s)	0.2	0.53	0.46	0.01	0.67	0.11	0.43	0.08	0.33	0.11	0.02	0.35	0.1	0.24	0	0.06	0.13	0.26
**2a.** Peak frequency of syllable A (Hz)	0.64	0.06	0.37	0.01	0.63	0.12	0.44	0.35	0.15	0.13	0.04	0.14	0.35	0.05	0.37	0.13	0.01	0.08
**3a.** Minimum frequency of syllable A (Hz)	0.53	0.17	0.6	0.07	0.31	0.2	0.71	0.13	0.23	0	0	0.28	0.4	0.25	0.41	0.22	0	0.46
**4a.** Maximum frequency of syllable A (Hz)	0.68	0.01	0.48	0.13	0.56	0.15	0.09	0.56	0.26	0.2	0.37	0	0.42	0.07	0.46	0.13	0.37	0.01
**5a.** Number of repeats of	--	--	0.09	0.04	0.33	0.13	0.44	0.06	0.31	0.03	0.07	0	0.01	0.04	0.08	0.01	--	--

syllable A																		
**6a.** Number of notes in syllable A	0.01	0.43	0.05	0	--	--	--	--	--	--	--	--	0.03	0.14	--	--	--	--
**8a.** Up-slurred or Down-slurred of syllable A (U or D)	--	--	0.03	0.15	--	--	--	--	--	--	--	--	--	--	--	--	--	--
**1a1.** Duration of note 1 of syllable A (s)	--	--	0	0.31	--	--	--	--	--	--	--	--	0.05	0	--	--	--	--
**2a1.** Peak frequency of note 1 of syllable A (s)	--	--	0.26	0.1	--	--	--	--	--	--	--	--	0.33	0.02	--	--	--	--
**3a1.** Minimum frequency of note 1 of syllable A (Hz)	--	--	0.26	0.19	--	--	--	--	--	--	--	--	0.25	0.04	--	--	--	--
**4a1.** Maximum frequency of note 1 of syllable A (Hz)	--	--	0.23	0.11	--	--	--	--	--	--	--	--	0.23	0.1	--	--	--	--
**8a1.** Up-slurred or Down-slurred of note 1 of syllable A (Hz)	--	--	0.03	0.31	--	--	--	--	--	--	--	--	0.03	0.1	--	--	--	--
**1a2.** Duration of note 2 of syllable A (s)	--	--	0.22	0.2	--	--	--	--	--	--	--	--	0.04	0	--	--	--	--
**2a2.** Peak frequency of note 2 of syllable A (s)	--	--	0.51	0.03	--	--	--	--	--	--	--	--	0.32	0.03	--	--	--	--
**3a2.** Minimum frequency of	--	--	0.63	0	--	--	--	--	--	--	--	--	0.23	0.09	--	--	--	--

note 2 of syllable A (Hz)																		
**4a2.** Maximum frequency of note 2 of syllable A (Hz)	--	--	0.57	0	--	--	--	--	--	--	--	--	0.16	0.03	--	--	--	--
**8a2.** Up-slurred or Down-slurred of note 2 of syllable A (Hz)	--	--	0	0.08	--	--	--	--	--	--	--	--	0.01	0.05	--	--	--	--
**1a3.** Duration of note 3 of syllable A (s)	--	--	0.22	0.01	--	--	--	--	--	--	--	--	--	--	--	--	--	--
**2a3.** Peak frequency of note 3 of syllable A (s)	--	--	0.25	0.41	--	--	--	--	--	--	--	--	--	--	--	--	--	--
**3a3.** Minimum frequency of note 3 of syllable A (Hz)	--	--	0.17	0.36	--	--	--	--	--	--	--	--	--	--	--	--	--	--
**4a3.** Maximum frequency of note 3 of syllable A (Hz)	--	--	0.15	0.45	--	--	--	--	--	--	--	--	--	--	--	--	--	--
**8a3.** Up-slurred or Down-slurred of note 3 of syllable A (Hz)	--	--	0.21	0.1	--	--	--	--	--	--	--	--	--	--	--	--	--	--
**1b.** Duration of syllable B (s)	0.33	0.27	0.12	0.11	0.03	0.34	0.02	0.03	0.09	0.09	0.11	0.44	0.14	0.46	0.34	0.03	0	0.02
**2b.** Peak frequency of syllable B (Hz)	0.67	0	0.29	0.31	0.64	0.12	0.34	0.24	0.02	0.19	0.05	0	0.4	0.18	0.21	0.03	0.27	0.08
**3b.** Minimum frequency of	0.59	0.08	0.32	0.22	0.63	0.03	0.06	0.34	0.06	0.17	0	0.14	0.43	0.16	0.33	0.01	0.05	0.2

syllable B (Hz)																		
**4b.** Maximum frequency of syllable B (Hz)	0.73	0.04	0.42	0.34	0.47	0.17	0.22	0.29	0.06	0.09	0.38	0.14	0.09	0.05	0.18	0.02	0.3	0.01
**5b.** Number of repeats of syllable B	--	--	0.25	0.07	0.01	0.19	0.44	0.01	0.02	0.08	0	0.18	0.43	0	0.26	0	--	--
**6b.** Number of notes in syllable B	0	0.15	0.49	0.02	--	--	--	--	--	--	--	--	0.05	0.05	--	--	--	--
**8b.** Up-slurred or Down-slurred of syllable B (U or D)	--	--	0.28	0.27	--	--	--	--	--	--	--	--	--	--	--	--	--	--
**1b1.** Duration of note 1 of syllable B (s)	--	--	0	0.03	--	--	--	--	--	--	--	--	0.03	0.23	--	--	--	--
**2b1.** Peak frequency of note 1 of syllable B (s)	--	--	0.03	0.43	--	--	--	--	--	--	--	--	0.33	0.19	--	--	--	--
**3b1.** Minimum frequency of note 1 of syllable B (Hz)	--	--	0.04	0.34	--	--	--	--	--	--	--	--	0.38	0.13	--	--	--	--
**4b1.** Maximum frequency of note 1 of syllable B (Hz)	--	--	0.06	0.44	--	--	--	--	--	--	--	--	0.11	0.06	--	--	--	--
**8b1.** Up-slurred or Down-slurred of note 1 of syllable B (Hz)	--	--	0.47	0.1	--	--	--	--	--	--	--	--	0.03	0	--	--	--	--
**1b2.** Duration of note 2 of syllable B (s)	--	--	0.01	0.04	--	--	--	--	--	--	--	--	--	--	--	--	--	--

**2b2.** Peak frequency of note 2 of syllable B (s)	--	--	0.15	0.11	--	--	--	--	--	--	--	--	--	--	--	--	--	--
**3b2.** Minimum frequency of note 2 of syllable B (Hz)	--	--	0.09	0.02	--	--	--	--	--	--	--	--	--	--	--	--	--	--
**4b2.** Maximum frequency of note 2 of syllable B (Hz)	--	--	0.13	0.09	--	--	--	--	--	--	--	--	--	--	--	--	--	--
**8b2.** Up-slurred or Down-slurred of note 2 of syllable B (Hz)	--	--	0.47	0.03	--	--	--	--	--	--	--	--	--	--	--	--		
**1c.** Duration of syllable C (s)	0.09	0.01	--	--	0.62	0.18	0.07	0.2	0.08	0.03	0.01	0.01	--	--	0.11	0.47	0.15	0.01
**2c.** Peak frequency of syllable C (Hz)	0.38	0.03	--	--	0.28	0.42	0.06	0.49	0.13	0.11	0.21	0.08	--	--	0.19	0.22	0.13	0.11
**3c.** Minimum frequency of syllable C (Hz)	0.59	0.08	--	--	0	0.37	0.06	0.11	0.11	0.13	0.34	0.13	--	--	0.2	0.55	0.02	0.24
**4c.** Maximum frequency of syllable C (Hz)	0.27	0.05	--	--	0.54	0.22	0.04	0.39	0.14	0.05	0.39	0.03	--	--	0.11	0.01	0.28	0
**5c.** Number of repeats of syllable C	--	--	--	--	0.5	0.16	0.06	0.05	0.03	0.02	0.25	0.1	--	--	0.03	0.09	--	--
**6c.** Number of notes in syllable C	0.36	0.02	--	--	--	--	--	--	--	--	--	--	--	--	--	--	--	--
**1d.** Duration of syllable D (s)	--	--	--	--	0.11	0.01	--	--	0.02	0.25	--	--	--	--	--	--	0.04	0.12

**2d.** Peak frequency of syllable D (Hz)	--	--	--	--	0.11	0.03	--	--	0	0.36	--	--	--	--	--	--	0.05	0.08
**3d.** Minimum frequency of syllable D (Hz)	--	--	--	--	0.02	0	--	--	0.01	0.33	--	--	--	--	--	--	0	0.43
**4d.** Maximum frequency of syllable D (Hz)	--	--	--	--	0.05	0.01	--	--	0	0.37	--	--	--	--	--	--	0.38	0
**5d.** Number of repeats of syllable D	--	--	--	--	0.16	0.08	--	--	0.03	0.01	--	--	--	--	--	--	--	--
**1e.** Duration of syllable E (s)	--	--	--	--	--	--	--	--	0.19	0.07	--	--	--	--	--	--	0.02	0.09
**2e.** Peak frequency of syllable E (Hz)	--	--	--	--	--	--	--	--	0.34	0.02	--	--	--	--	--	--	0.1	0
**3e.** Minimum frequency of syllable E (Hz)	--	--	--	--	--	--	--	--	0.15	0	--	--	--	--	--	--	0.02	0.1
**4e.** Maximum frequency of syllable E (Hz)	--	--	--	--	--	--	--	--	0.29	0.07	--	--	--	--	--	--	0.12	0.03
**5e.** Number of repeats of syllable E	--	--	--	--	--	--	--	--	0	0.01	--	--	--	--	--	--	--	--
**1f.** Duration of syllable F (s)	--	--	--	--	--	--	--	--	0.04	0	--	--	--	--	--	--	--	--
**2f.** Peak frequency of syllable F (Hz)	--	--	--	--	--	--	--	--	0.14	0.07	--	--	--	--	--	--	--	--
**3f.** Minimum frequency of syllable F (Hz)	--	--	--	--	--	--	--	--	0	0.09	--	--	--	--	--	--	--	--
**4f.** Maximum	--	--	--	--	--	--	--	--	0.12	0.01	--	--	--	--	--	--	--	--

frequency of syllable F (Hz)																		
**5f.** Number of repeats of syllable F	--	--	--	--	--	--	--	--	0.05	0	--	--	--	--	--	--	--	--
**1g.** Duration of syllable G (s)	--	--	--	--	--	--	--	--	0.05	0.08	--	--	--	--	--	--	--	--
**2g.** Peak frequency of syllable G (Hz)	--	--	--	--	--	--	--	--	0.35	0.18	--	--	--	--	--	--	--	--
**3g.** Minimum frequency of syllable G (Hz)	--	--	--	--	--	--	--	--	0.22	0.23	--	--	--	--	--	--	--	--
**4g.** Maximum frequency of syllable G (Hz)	--	--	--	--	--	--	--	--	0.38	0.14	--	--	--	--	--	--	--	--
**5g.** Number of repeats of syllable G	--	--	--	--	--	--	--	--	0.01	0.02	--	--	--	--	--	--	--	--
**1h.** Duration of syllable H (s)	--	--	--	--	--	--	--	--	0	0.01	--	--	--	--	--	--	--	--
**2h.** Peak frequency of syllable H (Hz)	--	--	--	--	--	--	--	--	0.04	0.02	--	--	--	--	--	--	--	--
**3h.** Minimum frequency of syllable H (Hz)	--	--	--	--	--	--	--	--	0	0.02	--	--	--	--	--	--	--	--
**4h.** Maximum frequency of syllable H (Hz)	--	--	--	--	--	--	--	--	0	0.04	--	--	--	--	--	--	--	--
**5h.** Number of repeats of syllable H	--	--	--	--	--	--	--	--	0.01	0	--	--	--	--	--	--	--	--
